# The der(1;7)(q10;p10) defining a distinct profile from −7/del(7q) in myelodysplastic syndromes: A systematic review and meta‐analysis

**DOI:** 10.1002/cam4.6890

**Published:** 2024-01-01

**Authors:** Wei Lang, Yingwan Luo, Lu Wang, Yudi Zhang, Chao Hu, Huanping Wang, Hongyan Tong

**Affiliations:** ^1^ Department of Hematology The First Affiliated Hospital of Zhejiang University Hangzhou China; ^2^ Zhejiang Provincial Key Laboratory of Hematopoietic Malignancy Zhejiang University Hangzhou China; ^3^ Zhejiang Provincial Clinical Research Center for Hematological disorders Hangzhou China; ^4^ Zhejiang University Cancer Center Hangzhou China

**Keywords:** cytogenetics, meta‐analysis, mutations, myelodysplastic syndrome

## Abstract

**Background and Objective:**

Myelodysplastic syndromes (MDS) are myeloid neoplasms characterized by ineffective hematopoiesis due to stem cell abnormalities. Monosomy 7q aberrations are a common cytogenetic abnormality in MDS. Specifically, an unbalanced translocation der(1;7)(q10;p10) [der(1;7)] has been identified in MDS patients, which is a monosomy 7q aberration variant like −7/del(7q). However, knowledge of der(1;7)'s features remains limited. Existing studies have compared the clinical and genetic characteristics of der(1;7) to those of −7/del(7q) but yielded inconsistent findings. Accordingly, we conducted meta‐analyses comparing der(1;7) to −7/del(7q).

**Methods:**

Publications were searched from the following databases up to January 10, 2023: Pubmed, Web of Science, Embase, Cochrane, and ClinicalTrials.gov. Eligible studies were assessed for risks of bias. Relevant data were extracted from included studies and analyzed using random‐effects models. Publication bias was evaluated and sensitivity analyses were performed.

**Results:**

The comparative meta‐analyses included 405 MDS patients with der(1;7) from nine studies. The analysis revealed that der(1;7) was associated with a greater male preponderance (86.1% vs. 68.3%, Odds Ratios (ORs) 2.007, *p* < 0.01) than −7/del(7q), lower platelets counts compared to del(7q), higher hemoglobin levels than −7, lower absolute neutrophil counts, and higher percentage of patients with non‐excess blasts (66.9% vs. 41.3%, ORs 2.374, *p* = 0.01) in comparison with −7/del(7q). The der(1;7) existed more as a sole karyotype aberration (55.6% vs. 37.0%, ORs 2.902, *p* = 0.02), co‐occurred more often with +8 (22.7% vs. 4.2%, ORs 5.714, *p* = 0.04) whereas less −5/del(5q) (1.5% vs. 41.3%, ORs 0.040, *p* < 0.01) and complex karyotype (7.3% vs. 54.8%, OR 0.085, *p* < 0.01). The der(1;7) was associated with higher frequencies of *RUNX1* (40.8% vs. 12.3%, ORs 4.764, *p* < 0.01), *ETNK1* (28.1% vs. 2.5%, ORs 42.106, *p* < 0.01) and *EZH2* (24.8% vs. 6.9%, ORs 3.767, *p* = 0.02) mutations, but less *TP53* mutation (2.4% vs. 45.3%, ORs 0.043, *p* < 0.01). Moreover, der(1;7) patients had longer time to progression (Hazard Ratios (HRs) 0.331, *p* = 0.02), better overall survival (OS) than −7 patients (HRs 0.557, *p* < 0.01), but similar OS with del(7q) patients (HRs 0.837, *p* = 0.37).

**Conclusion:**

The findings revealed distinct clinical, cytogenetic, and molecular characteristics distinguishing der(1;7) from −7/del(7q), indicating der(1;7) defines a unique subtype within MDS with monosomy 7q. These findings support classifying der(1;7) as a separate MDS entity in future.

## 1 INTRODUCTION

Myelodysplastic syndromes (MDS) are a group of clonal hematopoietic stem cell disorders characterized by clonal hematopoiesis with defective differentiation and abnormal proliferation, peripheral blood cytopenias, and a high risk of acute myeloid leukemia (AML) transformation.^1^ Chromosomal aberrations frequently occur in MDS,^2^ which contribute to the pathogenesis of MDS and are used for diagnoses, classification, treatment guidance, and prognostic stratification.^3^, ^4^, ^5^

The recently identified unbalanced translocation der(1;7)(q10;p10)[der(1;7)], a rare chromosomal abnormality seen in myeloid neoplasms,^6^, ^7^ involves fusion of the entire 1q arm to the 7p arm at the centromeric regions, resulting in monosomy 7q and 1q gain.^8^ Studies find der(1;7) in 0.58%–2.59% of myelodysplastic syndrome (MDS) cases,^7^, ^9^, ^10^ 0.2%–2% of acute myeloid leukemia (AML) cases, and about 1% of myeloproliferative neoplasms (MPN).^11^ Most der(1;7) cases are MDS (58%), followed by AML (26%) and MPN (9%). Furthermore, 80% of AML with der(1;7) are secondary or have myelodysplasia.^12^ Among MDS with der(1;7), some are therapy‐related MDS (t‐MDS), in varying proportions. Occasionally, rare phenotypes have been linked to this translocation, including MDS with eosinophilia (12 cases)^7^, ^13^, ^14^, ^15^, ^16^, ^17^ and organizing pneumonia (4 cases).^14^, ^18^

Studies have studied clinical features of der(1;7) by comparing them with those of −7/del(7q), which also involves monosomy 7q and occurs in around 10% of MDS cases predicting poor prognosis.^3^, ^6^, ^10^ However, these studies reported inconsistent results. Some studies could not confirm if der(1;7) more commonly appears as a sole karyotype compared to −7/del(7q).^6^, ^7^, ^19^, ^20^ Additionally, while Sanada et al.^6^ and Zhang et al.^7^ reported der(1;7) conferred a better prognosis in MDS, Slovak et al.^19^ and Hussain et al.^21^ suggested no significant survival difference between the two groups.

This study conducted a systematic review and meta‐analysis to summarize available data on: gender and age distribution, peripheral blood counts, bone marrow blast percentage, and spectrum of co‐occurring aberrations and mutations, prognosis between MDS with der(1;7) versus −7/del(7q). The aim was to overcome the limitations of prior smaller studies by analyzing pooled data on important clinical characteristics of MDS with der(1;7). This would assess differences between the two cytogenetic subgroups and help elucidate whether der(1;7) represents a unique clinical entity.

## 2 MATERIALS AND METHODS

### 2.1 Study registration and search strategy

This systematic review and meta‐analysis was registered with the International Prospective Register of Systematic Reviews (PROSPERO) (CRD42023387515). The methodology followed the Preferred Reporting Items for Systematic Reviews and Meta‐Analyses (PRISMA) 2020 statement guidelines.^22^ The following databases were searched for publications up to January 10, 2023: PubMed, Web of Science, Embase, Cochrane, and ClinicalTrials.gov. The search terms were liberalized as the followings to capture all relevant studies: (der(1;7) OR der(1;7)(q10;p10) OR der(7)t(1;7)(q10;p10)) AND (MDS OR (myelodysplastic syndromes) OR (myelodysplastic neoplasms)). Further details are provided in Supplementary Search Strategies.

### 2.2 Inclusion criteria and exclusion criteria

Studies were eligible for inclusion if they met the following criteria: studies that included patients detected with der(1;7)(q10;p10) and diagnosed with MDS, studies comparing relevant clinical features of MDS with der(1;7) versus −7/del(7q), studies providing sufficient information to calculate Odd Ratio (OR) for dichotomous variables or Hazard Ratio (HR) for survival with 95% confidence intervals (95% CI) for comparative analyses, and studies providing adequate statistical information on clinical features of der(1;7) for single‐arm meta‐analyses.

Studies were excluded if they were case reports or lacked sufficient quantitative data to enable comparative analyses or single‐arm meta‐analyses. Two authors independently screened studies for eligibility and resolved discrepancies by discussion.

### 2.3 Data extraction and risk of bias assessment

Data were extracted on author, journal/year, region, histopathology, cohort size and incidence of der(1;7), gender, age, percentage of t‐MDS/AML, peripheral blood counts, bone marrow blast percentage at diagnosis, frequencies of cytogenetic aberrations and mutations, and survival data for der(1;7) and −7/del(7q) MDS. Engauge Digitizer was applied to extract data from Kaplan–Meier curves to calculate HR and 95% CI using Tierney's method.^23^ The Newcastle‐Ottawa Scale (NOS) was used to assess study quality and risk of bias, with scores between 8 and 9 points indicating high quality with a low risk of bias, scores between 5 and 7 points indicating intermediate quality with an intermediate risk of bias, and scores less than 5 points indicating low quality with a high risk of bias.^24^

### 2.4 Statistical analysis

Comparative and single‐arm meta‐analyses were performed using either the Onlinemeta v1.0 web tool (https://smuonco.shinyapps.io/Onlinemeta/) or the “meta” and “metamedian” R packages.^25^, ^26^ Random effects models were utilized to calculate pooled estimates that account for within‐study random error and between‐study variation.^27^ A *p*‐value <0.05 indicated statistical significance. Cochrane *χ* ^2^ test and *I* ^2^ statistics were used to evaluate heterogeneity, with *I* ^2^ < 25%, 25%–50%, and >50% indicating low, moderate, and high heterogeneity, respectively.^28^ Publication bias was assessed using funnel plots, Egger's, and Begg's tests.^29^ Sensitivity analyses investigated the impact of removing individual studies on the pooled HRs for overall survival (OS) to evaluate the stability of the meta‐analysis.

## 3 RESULTS

### 3.1 Literature search

**FIGURE 1:**
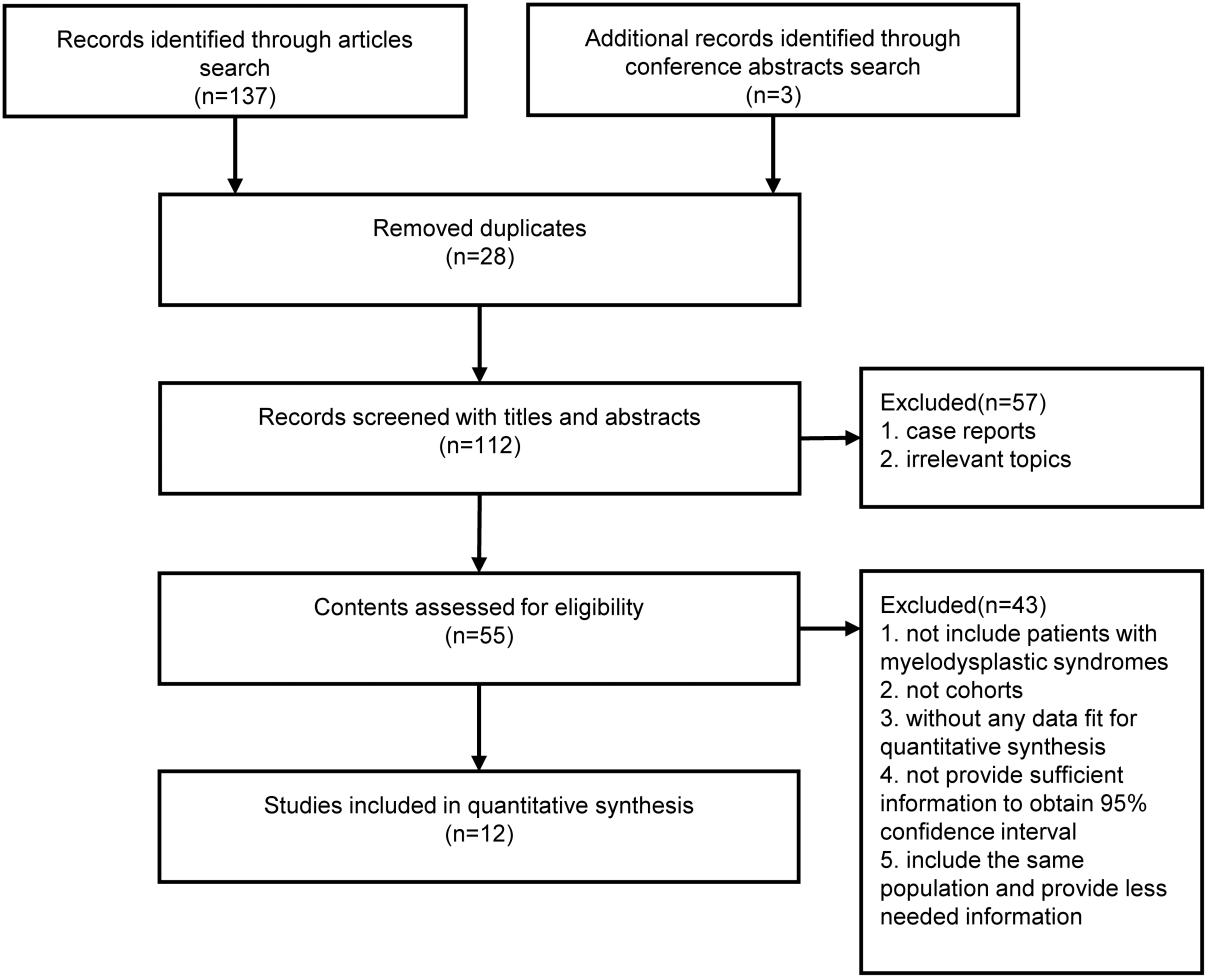
The flow diagram shows the selection process used to identify relevant studies for the review and meta‐analysis.

Two investigators independently searched databases, identifying 140 articles. After removing duplicates, 112 titles/abstracts were screened, excluding 57 unrelated studies. Of the remaining 55 articles assessed for eligibility, 12 studies were finally included in the quantitative synthesis. The search flow is shown in Figure 1.

### 3.2 Study characteristics and risk of bias assessment

The characteristics of the 12 eligible studies are shown in Table 1. The number of der(1;7) MDS patients ranged from 6 to 95 across the studies. A total of 405 MDS patients with der(1;7) from nine studies were included in both comparative and single‐arm meta‐analyses,^6^, ^7^, ^10^, ^13^, ^19^, ^20^, ^21^, ^30^, ^31^ and while three studies with 58 patients were only analyzed in single‐arm meta‐analyses.^8^, ^9^, ^32^ All were retrospective cohorts: 9 reported in articles, 2 in letters,^7^, ^9^ 1 in abstract.^13^ Five cohorts were from multiple centers.^6^, ^9^, ^10^, ^13^, ^20^ Of the 9 studies reported in articles, 3 had low and 6 intermediate risk of bias according to NOS (Table S1). The 2 from letters^7^, ^9^ and 1 from an abstract^13^ could not be assessed for NOS scores. However, these three studies all involved large cohort sizes and provided complete relevant clinical data. Due to these characteristics suggesting low or intermediate risk of bias, they were also included in the analytical investigations. No studies were determined to exhibit a high risk of bias in the analyses.

**TABLE 1:** The characteristic of der(1;7) MDS studies included in the meta‐analysis.

Study	Region	Pts Number	Diagnosis	MDS Subtype	Gender (M/F)	Age	Therapy‐related	Clinical variables used for comparative meta
Sanada, Leukemia, 2007^6^	Japan	64^a^	MDS(64/64)	RA(39/64) RAEB(9/64) RAEB‐t(6/64) CMML(4/64) MDS, NS(6/64)	57/7	67(17–88)	20/64	Gender, Blasts, Karyotype, Mutation, OS, TTP
Pozdnyako, Cancer, 2008^30^	USA	6^b^	MDS(6/6)	RCMD(‐RS)(2/6) RAEB(1/6) MDS‐U(3/6)	5/1	59(55–64)	NA	Gender, Blasts, OS
Slovak, Cancer Genetics and Cytogenetics,2009^19^	USA	12	MDS(6/12) Hypoplastic MDS(3/12) Cytogenetic abnormality only(1/12) NS(2/12)	RCMD(3/6) RAEB(2/6) RAEB‐t(1/6)	6/6	55.9(28–81)	4/12	Gender, Karyotype, OS
Hussain, Am. J. Hematol,2012^21^	USA	13	MDS(13/13)	RCUD(9/13) RARS(1/13) RCMD(1/13) RAEB(2/13)	11/2	62.4(13.1–87.0)	5/36^c^	Gender, Blasts, OS
Zhang, Leukemia, 2017[letter]^7^	China	50^d^	MDS(50/50)	RA/RN/RCUD(12/50) RCMD(24/50) RAEB‐1(9/50) RAEB‐2(5/50)	41/9	60(16–82)	NA	Gender, Blasts, Karyotype, Mutation, OS
Okuda, Blood, 2019[abstract]^13^	Japan and Germany	95^e^	MDS(84/95) AML(9/95) MPN(2/95)	NA	66/7	NA	NA	Mutation, Karyotype
Ganster, Genes Chromosomes Cancer, 2019^10^	Germany	63	MDS(61/63) CMML‐2(1/63) sAML(1/63)	RCUD(3/61) RA(RS)(7/61) RCMD(30/61) RAEB‐1(15/61) RAEB‐2(6/61)	57/8	66(22–87)	9/63	Gender, Blasts, OS, TTP
Itonaga, Bone marrow transplantation, 2019^31^	Japan	92	MDS(92/92)	RA(39/92) RAEB(43/92) RAEB‐t(10/92)	78/14	55.5(18–70)	0/92	Gender, Blasts, OS
Bernard, NEJM, 2022^20^	European and Japan	10	MDS(10/10)	MDS‐MLD(2/10) MDS‐EB(8/10)	7/3	62(53.25–69.5)	0/10	Gender, Blasts, Karyotype, Mutation
Wang, Blood, 2003^8^	Japan	20	MDS(20/20)	RA(14/20) RAEB(1/20) RAEB‐t(1/20) MDS, NS(6/20)	19/1	66(53.5–70)	7/20	Gender, Blasts but only included in the single‐arm analysis
Horio, eJHaem, 2020^32^	Japan	13	MDS(13/13)	MDS‐MLD(2/13) MDS‐EB‐1(3/13) MDS‐EB‐2(1/13) MDS‐U(7/13)	13/0	72(50–85)	1/13	Gender, Blasts but only included in the single‐arm analysis
Kozyra, Blood, 2021[Letter]^9^	European	25^f^	MDS(23/25) AML(2/25)	RCC(7/23) MDS‐MLD(6/23) MDS‐U(2/23) MDS‐EB(7/23) MDS, NS(1/23)	15/10	16(11–58)	NA	Gender, Blasts, and Mutation but only included in the single‐arm analysis

Abbreviations: Pts, patients; RA, refractory anemia; RARS, refractory anemia with ring sideroblasts; RAEB, refractory anemia with excess blasts; RAEB‐T, refractory anemia with excess blasts in transformation; CMML, chronic myelomonocytic leukemia; NS, not specific; OS, overall survival; TTP, time to acute myeloid leukemia (AML) progression; RCMD, refractory cytopenia with multilineage dysplasia; RS, ring sideroblasts; RCUD, refractory cytopenia with unilineage dysplasia; MDS‐U, myelodysplastic syndromes, unclassified; RN, refractory neutropenia; RCC, refractory cytopenia of childhood; sAML, secondary acute myeloid leukemia; MPN, MDS‐MLD, MDS with multilineage dysplasia; MDS‐SLD, MDS with single lineage dysplasia; NA, not available.

^a^ The der(1;7) incidence was 10/427 (2.3%) in the University of Tokyo Hospital MDS cohort.

^b^ The der(1;7) incidence was 6/1029 (0.58%) in the Rush and Umass MDS cohort.

^c^ Five therapy‐related MDS/AML patients along with 13 MDS, 2 AML, 11 MPN, 2 patients with non‐diagnostic results, 1 post‐MDS/MPN/AML patients, and 2 aplastic anemia/hypocellular MDS patients in Mayo Clinic cohorts.

^d^ The der(1;7) incidence was 50/1934 (2.59%) (9.6%) in the First Affiliated Hospital of Soochow University MDS cohort.

^e^ The der(1;7) incidence was 73/763 (0.80%) in the Japanese MDS cohort and 4/944 (0.42%) in the German MDS cohort.

^f^ The der(1;7) incidence was 13/1620 (0.80%) in Children/Adolescents primary MDS cohort enrolled in the European Working Group of MDS in Childhood registries.

### 3.3 The prevalence of der(1;7) in MDS

As shown in Figure S1, the pooled prevalence of der(1;7) in MDS was 2.2% (95% CI 1.2%–3.3%), varying between Asian and European/American regions (4.0%, 95% CI 1.4%–6.6% in Asia; 0.6%, 95% CI 0.3%–0.8% in Europe/America). The pooled frequency of therapy‐related cases was 20.4% (95% CI 11.6%–29.1%) among MDS with der(1;7).

### 3.4 Greater male preponderance of der(1;7) versus −7/del(7q)

**FIGURE 2:**
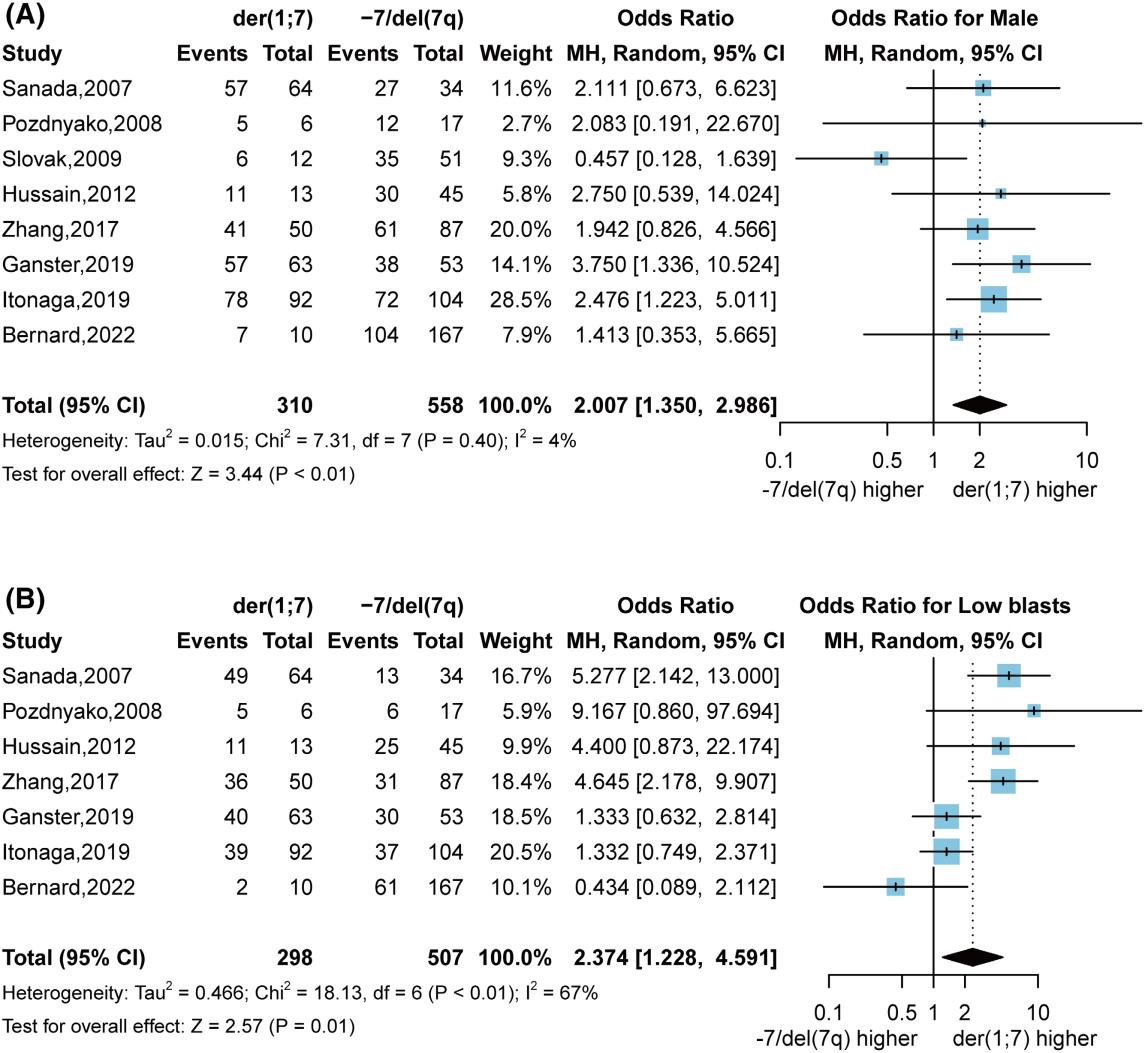
The pooled Odds Ratios (ORs) for males and MDS with low blasts in der(1;7) patients versus − 7/del(7q) patients. (A) The forest plot shows the pooled ORs for males. (B) The forest plot shows the pooled OR for MDS with low blasts in MDS patients with der(1;7) versus with −7/del(7q). The pooled ORs and their 95% confidence interval (95% CI) were calculated using the Mantel–Haenszel method with a random‐effects model. The size of each square represents the weight of that study in the meta‐analysis. The vertical dashed line shows the pooled ORs. And the vertical solid line represents an OR of 1. The diamond represents the pooled ORs and 95% CI. If they differ from 1, the difference is considered statistically significant.

The 12 studies provided information on the percentage of males in MDS with der(1;7) and eight studies provided data for −7/del(7q) (Figure S2). Both der(1;7) (86.1%, 95% CI 80.7% to 91.5%) and − 7/del(7q) MDS (68.3%, 95% CI 64.5%–72.2%) showed a male predominance. However, the pooled ORs for male gender (2.007, 95% CI 1.350–2.986, *p* < 0.01) indicated that der(1;7) had a significantly greater male predominance compared to −7/del(7q) (Figure 2A).

The median ages at diagnosis were 60.8 years (95% CI 55.4–69.5 years) for der(1;7) and 59.2 years (95% CI 52.3 to 66.1 years) for −7/del(7q) patients. There was no significant difference in median age between the two groups (pooled median difference 2.85 years, 95% CI −0.27 to 5.98 years, *p* = 0.07; Figure S3).

### 3.5 Lower platelets for der(1;7) versus del(7q) and higher hemoglobin for der(1;7) versus −7

The peripheral blood count data of der(1;7) and − 7/del(7q) patients are shown in Table 2. Patients with der(1;7) exhibited marked thrombocytopenia in six studies. The pooled median platelet counts were 69.5 × 10^9^/L (95% CI 57.4 × 10^9^/L to 81.7 × 10^9^/L) for der(1;7) patients, significantly lower than for del(7q) patients (median 118.3 × 10^9/^L; pooled median difference − 55.2 × 10^9/^L, 95% CI −99.6 × 10^9^/L to −10.8 × 10^9^/L, *p* = 0.0149) but similar to −7 patients (median 72.6 × 10^9/^L; pooled median difference 1.0 × 10^9^/L, 95% CI −30.7 × 10^9^/L to 32.7 × 10^9^/L, *p* = 0.2386).

**TABLE 2:** The peripheral blood counts of patients with der(1;7) and − 7 or del(7q) in MDS at diagnosis.

Study	Cytogenetics	Pts	WBC (*10^9^/L)	ANC (*10^9^/L)	HB (g/dL)	PLT (*10^9^/L)
Sanada, 2007^6^	der(1;7)	64	3.0 (0.8–34.0)	–	**9**.**0 (2**.**8**–**13**.7)*	**80 (13**–**870)**
	−7/del(7q)	34	2.4 (0.3–24.0)	–	7.8 (3.8–12.1)	81 (8–710)
Slovak, 2009^a^,^19^	der(1;7)	12	2.7 (0–6.8)	–	9.7 (8.1–11.4)	**57**.**5 (0**–**124)**
	−7	41	4.8 (2.9–6.8)	–	9.7 (9.0–10.4)	106 (75.7–136)
	del(7q)	10	4.8 (0.7–8.9)	–	10.4 (8.9–11.9)	146 (82.6–209)
Hussain, 2012^21^	sole der(1;7)	13	2.6	–	**11**.4*	**96**
	sole −7	30	3.1	–	8.9	77
	sole del(7q)	15	4.2	–	9.6	168
Zhang, 2017^7^	der(1;7)	50	**2**.**39 (1**.**01**–**7**.13)*	**1**.**26 (0**.**27**–**5**.75)*	**8**.**45 (3**.**1**–**13**.3)*	**64 (10**–**322)**
	−7/del(7q)	87	3.1 (1.15–16.8)	1.6 (0.16–13.29)	6.9 (3.2–13.7)	56 (6–561)
	non‐der(1;7)	1884	2.83 (1.2–10.8)	–	7.0 (1.1–16.7)	51(1–759)
Ganster, 2019^10^	sole der(1;7)	63	–	**1**.**03 (0**.**28**–**3**.29)*	**10**.**4 (4**.**7**–**14**.9)*	**77 (3**–432)*
	sole −7	41	–	1.63 (0.11–18.78)	9.1 (5.1–15.6)	58 (4–586)
	sole del(7q)	12	–	0.81 (0.34–16.43)	11.7 (7.5–13.7)	146 (28–496)
Bernard, 2022^20^	der(1;7)	10	2.75 (1.4–4.33)	0.768 (0.3–2.47)	**11 (7**.**6**–**13**.3)*	**62 (13**–**225)**
	−7/del(7q)	167	3 (0.6–11.1)	1.07 (0–8.632)	9 (5.3–15.8)	68 (5–448)
	−7	133	3 (1–11.1)	1.1 (0–8.632)	9 (5.3–15.8)	60 (5–448)
	del(7q)	34	3 (0.6–7.8)	0.95 (0–5.01)	8.85 (6.44–13.9)	95.5 (10–381)
Pooled results	der(1;7)	–	2.53 (2.16–2.90)^6^, ^7^, ^19^, ^20^	1.06 (0.90–1.22)^7^, ^10^, ^20^	9.6 (8.7–10.5)^6^, ^7^, ^10^, ^19^, ^20^	69.5 (57.4–81.7)^6^, ^7^, ^10^, ^19^, ^20^
	−7 or del(7q)	–	3.03 (2.77–3.29)^6^, ^7^, ^19^, ^20^	1.31 (0.95–1.69)^7^, ^10^, ^20^	9.1 (8.0–10.3)^6^, ^7^, ^10^, ^19^, ^20^	80.7 (59.6–101.9)^6^, ^7^, ^10^, ^19^, ^20^
	−7	–	–	–	9.2 (8.8–9.7)^10^, ^19^, ^20^	72.6 (44.2–100.9)^10^, ^19^, ^20^
	del(7q)	–	–	–	10.2 (8.5–11.9)^10^, ^19^, ^20^	118.3 (78.7–157.9)^10^, ^19^, ^20^
der(1;7) vs. −7/del(7q)	Δ, P of Δ	–	−0.45 (−0.99–0.10), 0.1100^6^, ^7^, ^19^, ^20^	**−0**.**33 (−0**.**67**–**0**.**00)**, **0**.**0495** ^7^, ^10^, ^20^	0.6 (−0.5–1.8), 0.2745^6^, ^7^, ^10^, ^19^, ^20^	3.9 (−22.9–15.1), 0.6867^6^, ^7^, ^10^, ^19^, ^20^
	*I* ^2^, *P* of *I* ^2^	–	0%, 0.5132	52%, 0.0915	60%, 0.0310	16%, 0.1564
der(1;7) vs −7	Δ, P of Δ	–	–	–	**1**.**2 (0**.**3**–**2**.**0)**, **0**.**0102** ^10^, ^19^, ^20^	1.0 (−30.7–32.7), 0.2386^10^, ^19^, ^20^
der(1;7) vs del(7q)	Δ, P of Δ	–	–	–	0.05 (−2.1–2.2), 0.9626^10^, ^19^, ^20^	**−55**.**2 (−99**.**6**–**10**.**8)**, **0**.**0149** ^10^, ^19^, ^20^

*Note*: Asterisks (*) indicate a *p*‐value <0.05, considered statistically significant differences as calculated by Bernard et al or extracted from other included studies. The pooled median estimates were calculated in ‘metamedian’ via the QE method. Δ, the pooled difference in median values. Bold values are used to emphasize some important data findings. The bold values in the ‘PLT’ column are intended to highlight that platelet counts of der(1;7) patients were generally lower than normal ranges. While the bold values without an asterisk(*) means there was no statistically significant difference between der(1;7) and −7/del(7q). P, *p*‐value. *I* ^2^, total heterogeneity/total variability.

Abbreviations: ANC, absolute neutrophil counts; HB, hemoglobin; Pts, patients; PLT, platelets; WBC, white blood cells.

^*a*^ Data presented as mean (95% confidence interval) in Slovak et al while median (range) for other studies.

The pooled median hemoglobin (HB) was 9.6 g/dL (95% CI 8.7 g/dL to 10.5 g/dL) for der(1;7), higher than for −7 patients (median 9.2 g/dL; pooled median difference 1.2 g/dL, 95% CI 0.3 g/dL to 2.0 g/dL, *p* = 0.0102) but similar to del(7q) patients (median 10.2 g/dL; pooled median difference 0.05 g/dL, 95% CI −2.1 g/dL to 2.2 g/dL, *p* = 0.9626).

The pooled median absolute neutrophil counts (ANC) were 1.06 × 10^9^/L (95% CI 0.90 × 10^9^/L to 1.22 × 10^9^/L) for der(1;7) and 1.31 × 10^9^/L (95% CI 0.95 × 10^9^/L to 1.69 × 10^9^/L) for −7/del(7q) patients. And der(1;7) patients had a lower ANC than −7/del(7q) patients (pooled median difference − 0.33 × 10^9^/L, 95% CI −0.67 × 10^9^/L to −0.0008 × 10^9^/L, *p* = 0.0495).

### 3.6 Higher percentage of MDS with low blasts in der(1;7) versus −7/del(7q)

The pooled percentage of MDS with low blasts (<5% myeloblasts) was 66.9% (95% CI 55.1%–78.7%) for 350 der(1;7) patients from 10 cohorts, and 41.3% (95% CI 34.6% to 48.0%) for 507–7/del(7q) patients from 7 cohorts (Figure S4).

The der(1;7) showed higher odds of low blast MDS compared to −7/del(7q) (ORs 2.374, 95% CI 1.228 to 4.591, *p* = 0.01), indicating that der(1;7) is more commonly associated with non‐excess blasts at diagnosis (Figure 2B).

### 3.7 Comparisons on co‐occurring cytogenetic aberrations

The pooled frequencies of co‐occurring cytogenetic aberrations with der(1;7) were calculated from 4 non‐overlapping cohorts and 157 der(1;7) MDS cases from the Mitelman database^12^ (Figure S5). Frequencies of co‐occurring aberrations were also determined for 351 MDS cases with −7/del(7q) from the four cohorts mentioned above (Figure S6).

**FIGURE 3:**
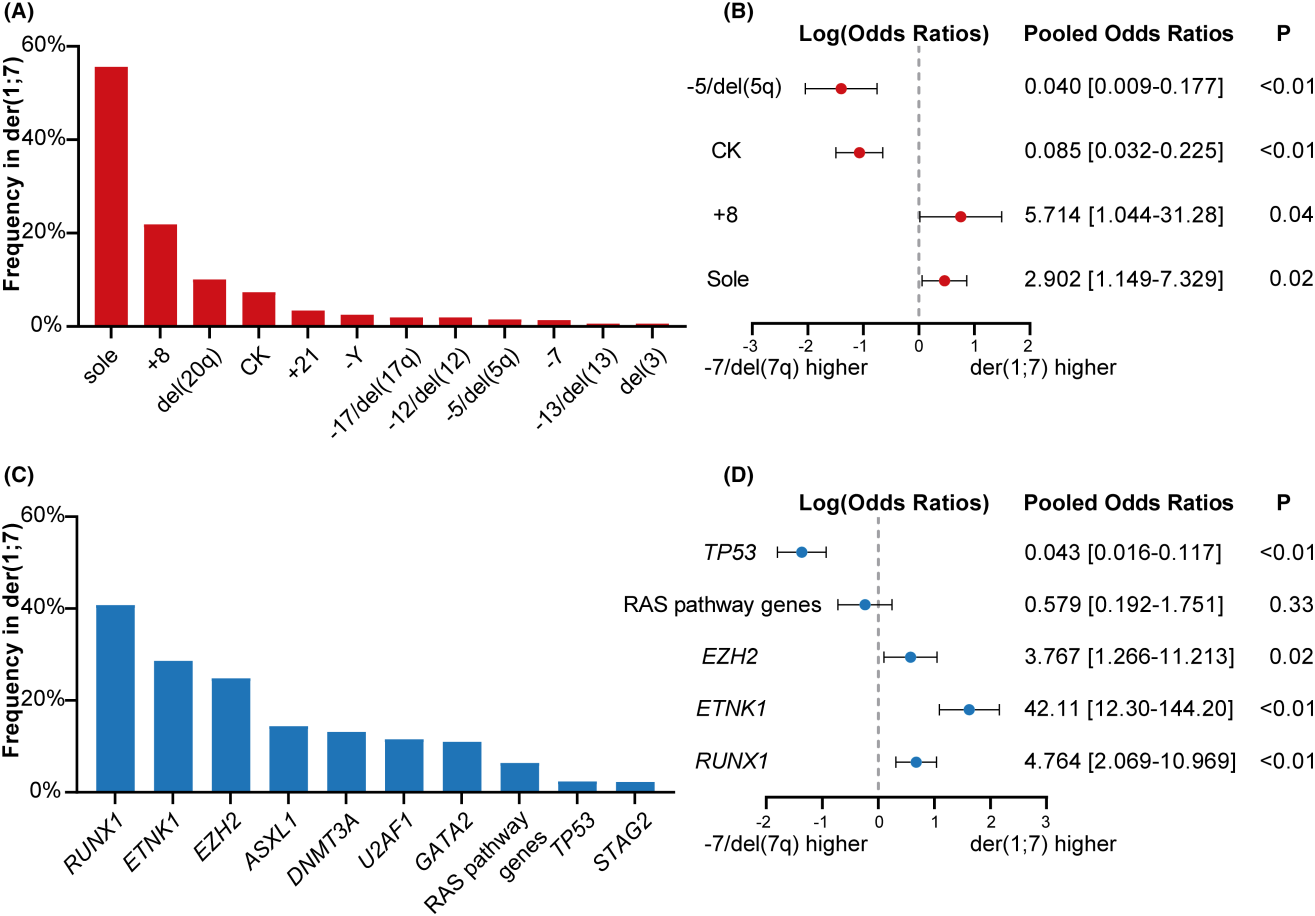
The cytogenetic and mutational profiles of der(1;7) patients and the pooled Odds Ratios (ORs) for co‐lesions in der(1;7) versus − 7/del(7q) patients. (A) The pooled frequencies of co‐occurring cytogenetic aberrations in der(1;7) patients. (B) The pooled ORs for sole chromosomal aberration, +8, complex karyotype and − 5/del(5q) in der(1;7) versus −7/del(7q) patients. (C) The pooled mutation frequencies in der(1;7) patients. (D) The pooled ORs for *RUNX1*, *ETNK1*, *EZH2*, *TP53* and RAS pathway gene mutation in der(1;7) versus −7/del(7q) patients. RAS pathway genes include *CBL*, *KRAS*, *NRAS*, *NF1* and *PTPN11*. The pooled frequencies were calculated using single‐arm meta‐analysis with an inverse variance random effects model. The pooled ORs were calculated using Mantel–Haenszel random effects meta‐analysis. The dot represents the pooled ORs, and the error bar represents 95% confidence interval. The x‐axis is on a log scale. The vertical dashed line represents an OR of 1. A *p*‐value <0.05 is considered statistically significant.

The der(1;7) occurred as a sole karyotype abnormality at diagnosis in 55.6% (95% CI 49.2% to 62.0%) of patients with der(1;7). Common co‐occurring aberrations in der(1;7) patients were + 8 (22.7%, 95% CI 18.0% to 27.3%), del(20q) (10.1%, 95% CI 3.1% to 17.2%), followed by +21 (3.4%, 95% CI 1.4% to 8.1%), −5/del(5q) (1.5%, 95% CI 0.0% to 3.1%), and −7 (1.4%, 95% CI 0.0% to 3.1%). Complex karyotype (CK) occurred in 7.3% (95% CI 3.9% to 10.8%) of der(1;7) patients (Figure 3A).

However, only 4.8% (95% CI 0.0% to 10.2%) of −7/del(7q) cases co‐occurred with +8. Meanwhile, 37.0% (95% CI 18.1% to 56.0%) of −7/del(7q) cases occurred as a sole aberration. In contrast, 54.8% (95% CI 48.6% to 60.9%) of −7/del(7q) patients had CK and 41.3% (95% CI 35.7% to 46.8%) showed −5/del(5q) co‐occurrence.

Compared to −7/del(7q), der(1;7) more often occurred as a sole aberration (ORs 2.902, 95% CI 1.149 to 7.329, *p* = 0.02), while co‐occurring with +8 more frequently (ORs 5.714, 95% CI 1.044 to 31.28, *p* = 0.04). Patients with der(1;7) had less frequently CK (ORs 0.085, 95% CI 0.032 to 0.225, *p* < 0.01) and − 5/del(5q) (ORs 0.040, 95% CI 0.009 to 0.177, *p* < 0.01) compared to those with −7/del(7q) (Figure 3B and Figure S7).

### 3.8 Comparisons on mutational profiles

Mutational data was examined for 157 der(1;7) patients (Figure S8). The common mutated genes in der(1;7) patients and their frequencies were as follows: *RUNX1* (40.8%, 95% CI 32.8% to 48.7%), *ETNK1* (28.1%, 95% CI 19.8% to 36.3%), *EZH2* (24.8%, 95% CI 17.3% to 32.3%), *GATA2* (11% in adult but 72.7% in pediatric MDS). Other mutated genes were *ASXL1* (14.4%), *DNMT3A* (13.2%), *U2AF1* (11.6%), RAS pathway genes (6.4%, 95% CI 0.0% to 15.7%, including *CBL*, *KRAS*, *NRAS*, *NF1* and *PTPN11* ^13^), *TP53* (2.4%, 95% CI 0.0% to 5.6%), and *STAG2* (2.3%, 95% CI 0.0% to 5.1%) (Figure 3C and Figure S9).

Mutation data was also analyzed for 351–7/del(7q) patients (Figure S9). The most common mutations in −7/del(7q) patients were *TP53* (45.3%, 95% CI 33.4% to 57.1%), RAS pathway genes (18.5%, 95% CI 13.4% to 23.7%), followed by *RUNX1* (12.3%, 95% CI 7.9% to 16.6%). *EZH2* (6.9%, 95% CI 3.4% to 10.5%) and *ETNK1* (2.5%, 95% CI 0.0% to 5.5%) mutations were observed at relatively low frequencies.

In comparison, der(1;7) often accompanies *RUNX1* mutations, and the frequency of *RUNX1* mutations was significantly higher in der(1;7) than in −7/del(7q) (ORs 4.764, 95% CI 2.069 to 10.969, *p* < 0.01). The mutation frequencies of *EZH2* (ORs 3.767, 95% CI 1.266 to 11.213, *p* = 0.02) and *ETNK1* (ORs 42.106, 95% CI 12.295 to 144.2, *p* < 0.01) were also significantly higher in der(1;7), whereas *TP53* mutation frequency (ORs 0.043, 95% CI 0.016 to 0.117, *p* < 0.01) was lower, suggesting distinct mutation profiles between the two cytogenetic groups (Figure 3D and Figure S10).

### 3.9 Better OS for der(1;7) versus −7 but similar OS of der(1;7) with del(7q)

**FIGURE 4:**
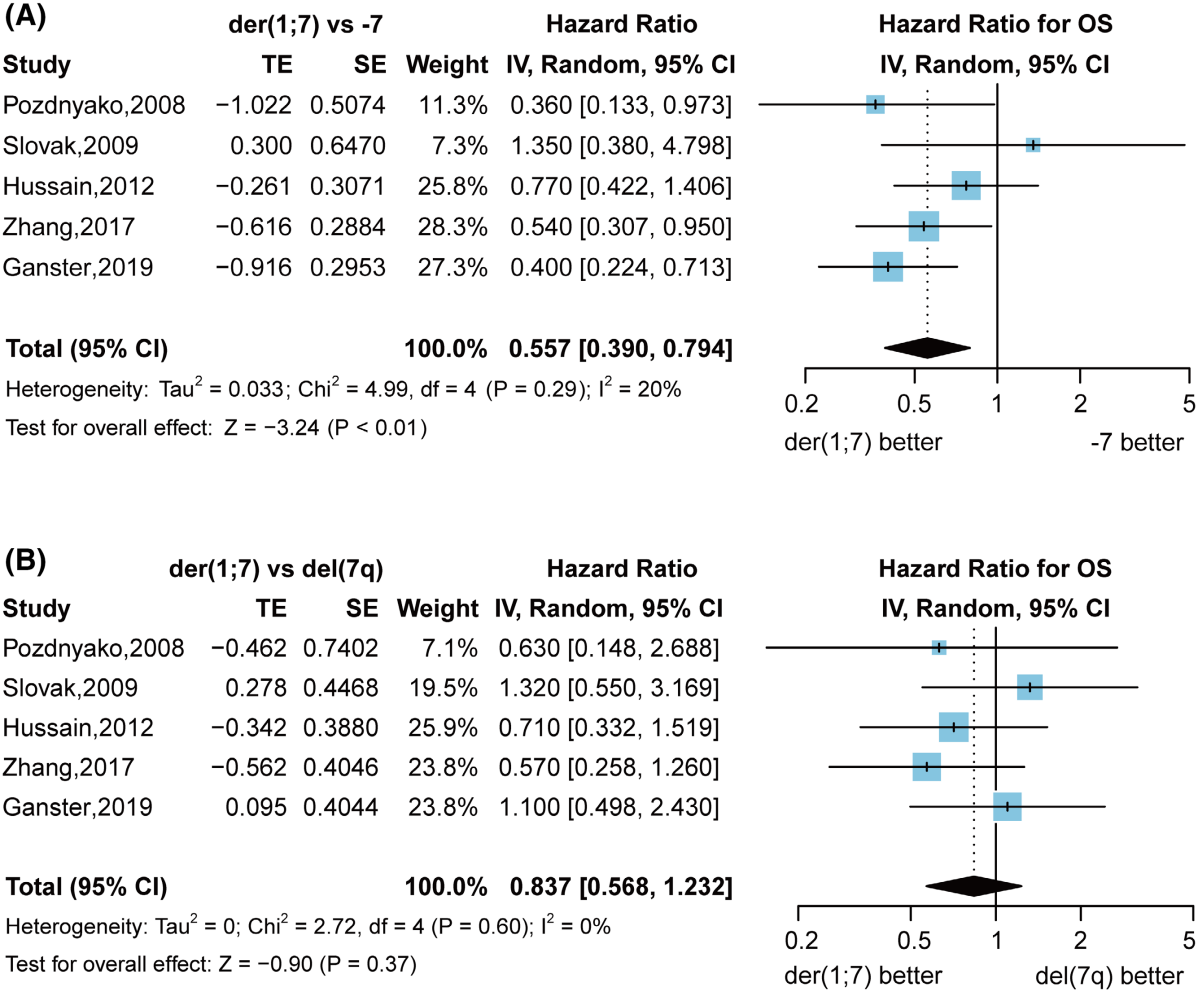
Better Overall Survival (OS) for der(1;7) versus − 7 and similar OS for der(1;7) and del(7q) patients. (A) The forest plot shows the Hazard Ratios (HRs) of OS in patients with der(1;7) versus −7 (B) The forest plot shows the HRs of OS in patients with der(1;7) versus del(7q). The pooled HRs were calculated using the inverse variance method with a random effects model. The diamond represents the pooled HRs and 95% CI. And the vertical solid line represents a HR of 1. If the pooled HRs and its 95% confidence interval (95% CI) are <1, indicating a lower risk of death for der(1;7) patients. If the pooled HRs and 95% CI crosses 1, indicating no significant difference in OS.

The pooled HRs for OS comparing der(1;7) and non‐dividing −7/del(7q) patients were 0.443 (95% CI 0.229 to 0.856, *p* = 0.02) with high heterogeneity (Figure S11). Subgroup analyses were performed by comparing OS for der(1;7) with −7 and der(1;7) with del(7q) separately, which mitigated the heterogeneity (Figure 4). The pooled HRs for overall survival (OS) comparing der(1;7) versus −7 patients were 0.557 (95% CI 0.390 to 0.794, *p* < 0.01), indicating better OS for der(1;7) patients compared to −7 patients. However, no significant difference in OS was observed between der(1;7) and del(7q) patients (HRs 0.837, 95% CI 0.568 to 1.232, *p* = 0.37).

Regarding the prognostic difference between sole der(1;7) and sole −7 patients, the overall HRs from two cohorts for OS were insignificantly 0.552 (95% CI 0.291 to 1.05, *p* = 0.0695) under random effects models but significantly 0.548 (95% CI 0.361 to 0.8315, *p* < 0.01) under fixed effects models. Upon including another study that compared sole der(1;7) and sole −7/del(7q) patients in the meta‐analysis,^31^ the pooled HRs were significant under both fixed (0.614, 95% CI 0.453 to 0.833, *p* < 0.01) and random effects models (0.608, 95% CI 0.417 to 0.888, *p* < 0.01; Figure S12).

The pooled HRs for time to AML progression were 0.331 (95% CI 0.128 to 0.856, *p* = 0.02), suggesting slower progression for der(1;7) versus −7/−7q patients (Figure S13).

### 3.10 Sensitivity analysis and publication bias analysis

**FIGURE 5:**
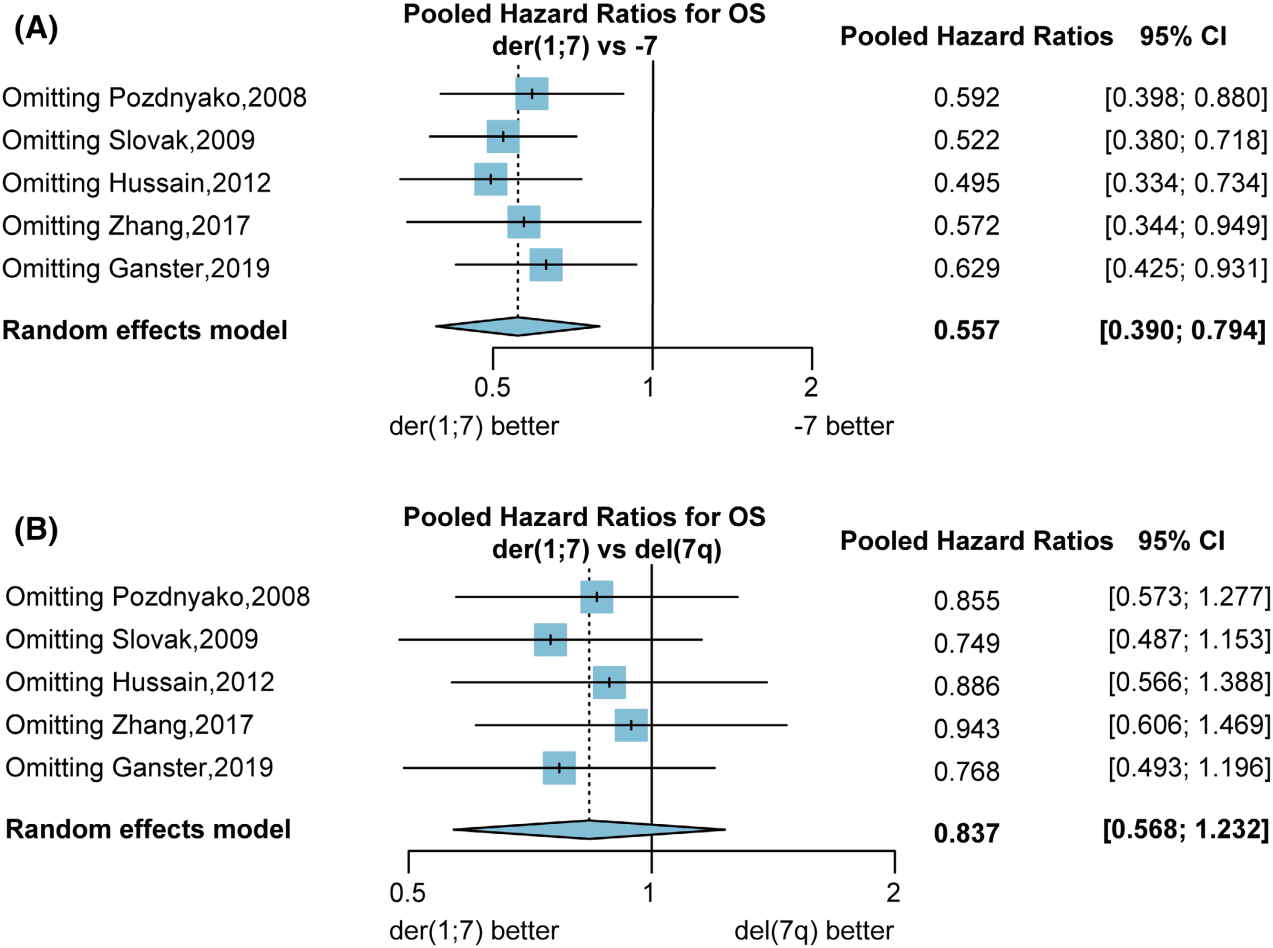
Sensitivity analyses evaluating the stability of the results. (A) The sensitivity analysis of the pooled Hazard Ratios (HRs) for overall survival (OS) in der(1;7) versus −7. (B) The sensitivity analysis of the pooled HRs for OS in der(1;7) versus del(7q). For the sensitivity analysis, the pooled HRs and 95% confidence interval of OS were recalculated after excluding each study individually.

A sensitivity analysis was conducted to verify the robustness of the meta‐analysis findings on OS difference between der(1;7) with −7 and der(1;7) with del(7q). Excluding any single study had little effect on the pooled results, indicating the stability of the findings (Figure 5). The funnel plots for OS appeared approximately symmetric, suggesting no substantial publication bias (Figure S14). Furthermore, Egger's test and Begg's test revealed no evidence of publication bias for all the comparative meta‐analyses (Table S2).

## 4 DISCUSSION

Myelodysplastic syndromes (MDS) are characterized by ineffective hematopoiesis and high genetic heterogeneity. In the 2022 WHO classification of MDS,^33^ MDS‐5q, MDS‐*SF3B1*, and MDS‐bi*TP53* have been classified as distinct subtypes based on their specific genetic features and associated clinical characteristics. Recently, more additional abnormal karyotypes and their corresponding clinical phenotypes have been identified in MDS patients.^34^, ^35^ The −7/del(7q) and der(1;7) abnormalities are both characterized by loss of 7q. The −7/del(7q) abnormality is relatively common in MDS and is associated with increased myeloblasts and poor prognosis,^3^ while der(1;7) is less common and its phenotypes in MDS are not well summarized. Here, through meta‐analysis, we identified a distinct clinical and genetic profile of MDS patients with der(1;7) compared to patients with −7/del(7q). The der(1;7) is characterized by a greater male preponderance, marked thrombocytopenia, higher HB, lower ANC, and lower blasts in MDS, with more sole aberration existence and co‐occurrence of +8 in karyotype, and with higher frequencies for *RUNX1*, *ETNK1*, and *EZH2* mutations. In contrast, the der(1;7) co‐occurred infrequently with −5/del(5q), CK, and *TP53* mutations. Additionally, der(1;7) transformed to AML more slowly than −7 or del(7q) patients, showed longer OS compared with −7 patients, and had a similar OS with del(7q) patients. Our findings support categorizing der(1;7) as a unique entity within the group of MDS with monosomy 7q.

Persistent cytopenia in one or more peripheral blood cell lineages is a key diagnostic criterion for MDS,^1^ and our analysis demonstrated significant differences in blood counts among patients with der(1;7), −7, and del(7q). Studies included in our meta‐analysis indicated that marked thrombocytopenia and coexisting anemia of varying severity were prominent clinical features in MDS with der(1;7). The meta‐analysis also further revealed that patients with der(1;7) had significantly lower platelet counts compared to patients with del(7q) and higher hemoglobin levels compared to patients with −7. The study by Hussain et al. showed a similar trend that corroborated our findings.^21^ it. Absolute neutrophil counts were compared in only two studies. Therefore, to strengthen our analysis, we additionally included raw data from Bernard et al.'s cohort.^20^ With this extra data, our meta‐analysis found that median absolute neutrophil counts were decreased in patients with der(1;7) and even lower compared to patients with −7/del(7q). RNA sequencing also suggested innate immunity pathways were more downregulated in der(1;7) patients versus −7/del(7q) and other karyotypes.^7^, ^36^ These factors may partly explain increased odds of infections in der(1;7) cases.^36^

Our analysis revealed distinct cytogenetic and mutational profiles, and several findings provide evidence suggesting that der(1;7) represent an initiating or early clone in MDS. First, in nearly half of patients with der(1;7) at diagnosis, der(1;7) exhibited as the sole chromosomal abnormality, without other chromosomal aberrations. Second, three sole der(1;7) MDS cases even lacked common somatic mutations of MDS,^20^, ^37^ suggesting der(1;7) can be an initiating clone independent from other drivers of MDS. Third, we found that patients with der(1;7) had high frequencies of +8, and karyotype analysis demonstrated that +8 arose as a subclone from the original der(1;7) clone in some cases,^12^ indicating that der(1;7) occurs earlier. *EZH2*, located at 7q36.1, was mutated more commonly in der(1;7) patients, and its mutations were also likely acquired later after the major der(1;7) clones based on mutation burden.^13^

Thrombocytopenia in der(1;7) patients may be partially due to transcription factor dysregulation. During normal hematopoiesis, GATA1/2 interacts with FOG‐1 to regulate early megakaryopoiesis.^38^ RUNX1 bifurcates the erythroid and megakaryocytic lineages, inhibits erythropoiesis while favors megakaryopoiesis,^39^ and is also crucial for megakaryocyte terminal maturation.^40^ However, a study found hypermethylation of enhancer regions associated with GATA1‐3 and RUNX1 binding sites in patients with der(1;7),^37^ indicating dysregulation of these transcription factors at the epigenetic level. Concurrently, our meta‐analysis confirmed a high frequency of *RUNX1* mutations in patients with der(1;7). Both the epigenetic changes and *RUNX1* mutations likely result in abnormal RUNX1 regulation in der(1;7) MDS, potentially influencing normal megakaryocyte development.

The study also revealed other significant differences in co‐occurring genetic alterations between patients with der(1;7) and − 7/del(7q). *ETNK1* mutations were enriched in der(1;7) patients, and their combinations may be potentially associated with MDS with eosinophilia,^13^ warranting further research into this specific phenotype. In contrast, *TP53* mutations, complex karyotype, and − 5/del(5q) abnormalities indicative of poorer prognosis in MDS with monosomy 7q,^41^ were more frequent in patients with −7/del(7q) while less frequent in patients with der(1;7).

In contrast to patients with −7/del(7q) who tend to progress to advanced MDS,^3^, ^10^, ^42^ we found patients with der(1;7) had lower myeloblasts and slower transformation to AML. We also found that der(1;7) patients exhibited longer OS compared to −7/del(7q) patients, though with high heterogeneity. Our subgroup analysis reduced the heterogeneity and further clarified that der(1;7) patients had longer OS than −7 patients but similar survival as del(7q) patients in MDS. Sensitivity analysis confirmed the stability of this conclusion.

Notably, two studies yielded inconsistent results regarding the prognosis of patients with sole der(1;7) compared to those with sole −7.^10^, ^21^ One suggested no difference in OS while the other showed longer OS for sole der(1;7). The meta‐analysis of the two studies using different statistical model also showed inconsistent findings. The random‐effects model suggested a favorable but insignificant trend for sole der(1;7), while the fixed‐effects model showed better OS for sole der(1;7). This inconsistency may due to the limited number of studies and the wider confidence intervals with random effects models.^27^ More relevant studies are needed to address differences in OS between sole der(1;7) and sole −7 in MDS. Considering the survival characteristics of −7 may be similar to those of −7/del(7q), as −7 accounted for around 80% of −7/del(7q),^43^ we included another study finding that sole der(1;7) had significantly longer OS and relapse time after transplantation than sole −7/del(7q).^31^ Combining data from the three studies, both fixed and random‐effects models corroborate better prognosis for sole der(1;7) compared to sole −7.

This study has some limitations. First, the inclusion of only retrospective cohort studies introduces potential for selection bias, information bias, and confounding. Second, some studies did not adjust for potential confounders like age, sex, and disease risk in outcome analyses. Prognostic differences may be influenced by these factors. Third, two studies reported that der(1;7) patients could benefit more from transplantation with longer relapse time and improved survival,^19^, ^31^ however, the limited research precluded further subgroup analyses by specific treatment regimens. Studies focusing on response to specific therapies are further warranted.

To our knowledge, this is the first meta‐analysis comparing the clinical and genetic characteristics of der(1;7) versus −7/del(7q). The study primarily found that der(1;7) was associated with a greater male preponderance, lower blasts, lower platelet counts than del(7q), higher hemoglobin than −7. Our study highlighted differences in cytogenetic abnormalities and mutations between the two groups. Moreover, der(1;7) also exhibited a longer time to AML progression, improved OS versus −7, and similar OS as del(7q) in MDS. In summary, this study resolved inconsistencies in previous studies by analyzing pooled data and identified key clinical characteristics that differentiate MDS patients with der(1;7) from those with −7/del(7q). Our findings provide preliminary evidence to consider classifying der(1;7) as a distinct MDS subtype in the future.

## AUTHOR CONTRIBUTIONS

**Wei Lang:** Conceptualization (equal); data curation (equal); formal analysis (equal); investigation (equal); methodology (equal); project administration (equal); resources (equal); software (equal); writing – original draft (equal); writing – review and editing (equal). **Yingwan Luo:** Conceptualization (equal); methodology (equal); project administration (equal); resources (equal); writing – review and editing (equal). **Lu Wang:** Writing – review and editing (equal). **Yudi Zhang:** Supervision (equal); writing – review and editing (equal). **Chao Hu:** Methodology (equal); writing – review and editing (equal). **Huanping Wang:** Methodology (equal). **Hongyan Tong:** Funding acquisition (lead); supervision (lead).

## FUNDING INFORMATION

This work was supported by the National Natural Science Foundation of China (81970117) to Hongyan Tong.

## CONFLICT OF INTEREST STATEMENT

No potential conflict of interest was reported by the authors.

## ETHICS STATEMENT

It is exempt from requiring an ethics approval statement as this study is a meta‐analysis. Meta‐analyses do not involve directly collecting new data from human participants, so informed consent is also not applicable here.

## Supporting information


Appendix S1.

## Data Availability

The data used to calculate the results of this study are included within the article and supplement.
