# Supplementary material for: The der(1;7)(q10;p10) defining a distinct profile from −7/del(7q) in myelodysplastic syndromes: A systematic review and meta‐analysis
Source: Cancer Med. 2024 Jan 1;13(1):e6890. doi: 10.1002/cam4.6890 (PMC10807610; doi:10.1002/cam4.6890)
Supplement: Supplementary file 1 — Appendix S1. [file CAM4-13-e6890-s001.docx]

**Supplement Search Strategies.**

The following databases were searched for publications up to January 10, 2023: Web of Science, Embase, PubMed, Cochrane, and ClinicalTrials.gov. To capture all relevant studies on MDS with der(1;7), we developed a comprehensive search strategy. The terms (der(1;7) OR der(1;7)(q10;p10) OR der(7)t(1;7)(q10;p10)) AND (MDS OR (Myelodysplastic syndromes) OR (Myelodysplastic neoplasms)) were used initially. If search results were limited, the terms were expanded to simply der(1;7). Additionally, reference lists of key publications were reviewed for other relevant studies. The aim was to identify as many cases of MDS with der(1;7) as possible through a systematic literature search.

| **Database and Searches** | **Results** |
| --- | --- |
| Web of Science:  (TS=(der(1;7)) OR TS=(der(1;7)(q10;p10)) OR TS=(der(7)t(1;7)(q10;p10))) AND (TS=(MDS) OR TS=(Myelodysplastic syndromes) OR TS=(Myelodysplastic neoplasms)) | 51 |
| Embass:  #1 'der(1;7)' OR 'der(1;7)(q10;p10)' OR 'der(7)t(1;7)(q10;p10)'  #2 mds OR (myelodysplastic AND syndromes) OR (myelodysplastic AND neoplasms)  #1 AND #2 | 82 |
| PubMed:  der(1;7) | 4 |
| Cochrance:  der(1;7) in Title Abstract Keyword OR der(1;7)(q10;p10) in Title Abstract Keyword OR der(7)t(1;7)(q10;p10) in Title Abstract Keyword | 0 |
| ClinicalTrials.gov:  der(1;7) | 0 |

**Supplement Figure S1. The pooled prevalence of der(1;7) in myelodysplastic syndrome (MDS) and the frequency of therapy-related MDS in MDS with der(1;7).** (A) The variability in der(1;7) prevalence between Asian and European/American MDS patients. (B) The pooled frequency of therapy-related cases among MDS with der(1;7). The random-effect model with the inverse variance method are utilized in both single-arm meta-analyses.


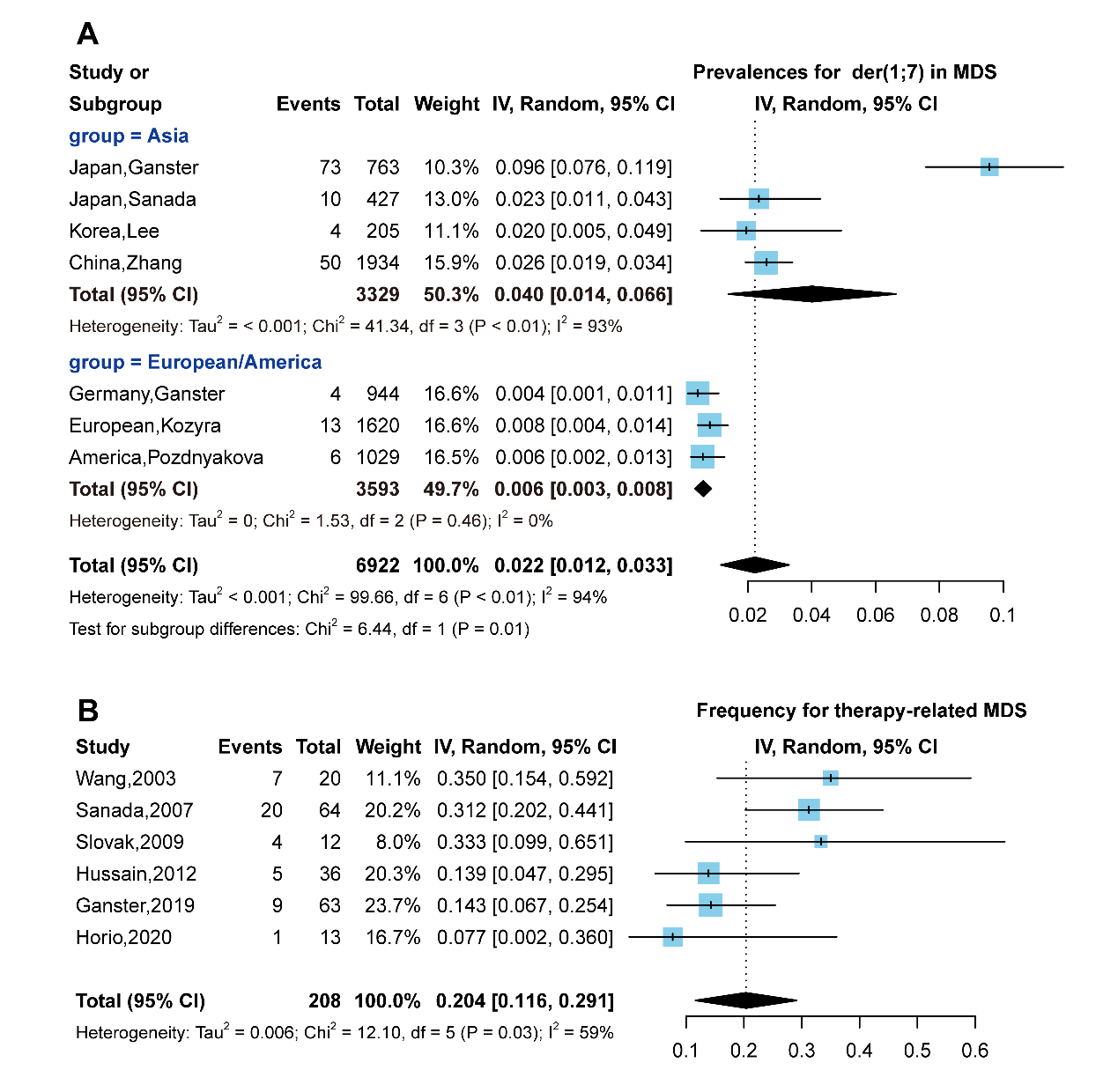


**Supplement Figure S2. The pooled proportions for male gender in myelodysplastic syndrome (MDS) patients with der(1;7) and -7/del(7q).** (A) The forest plot shows the pooled proportion of male patients in MDS with der(1;7). (B) In MDS with -7/del(7q). Both single-arm meta analyses use the random-effect model with the inverse variance method.


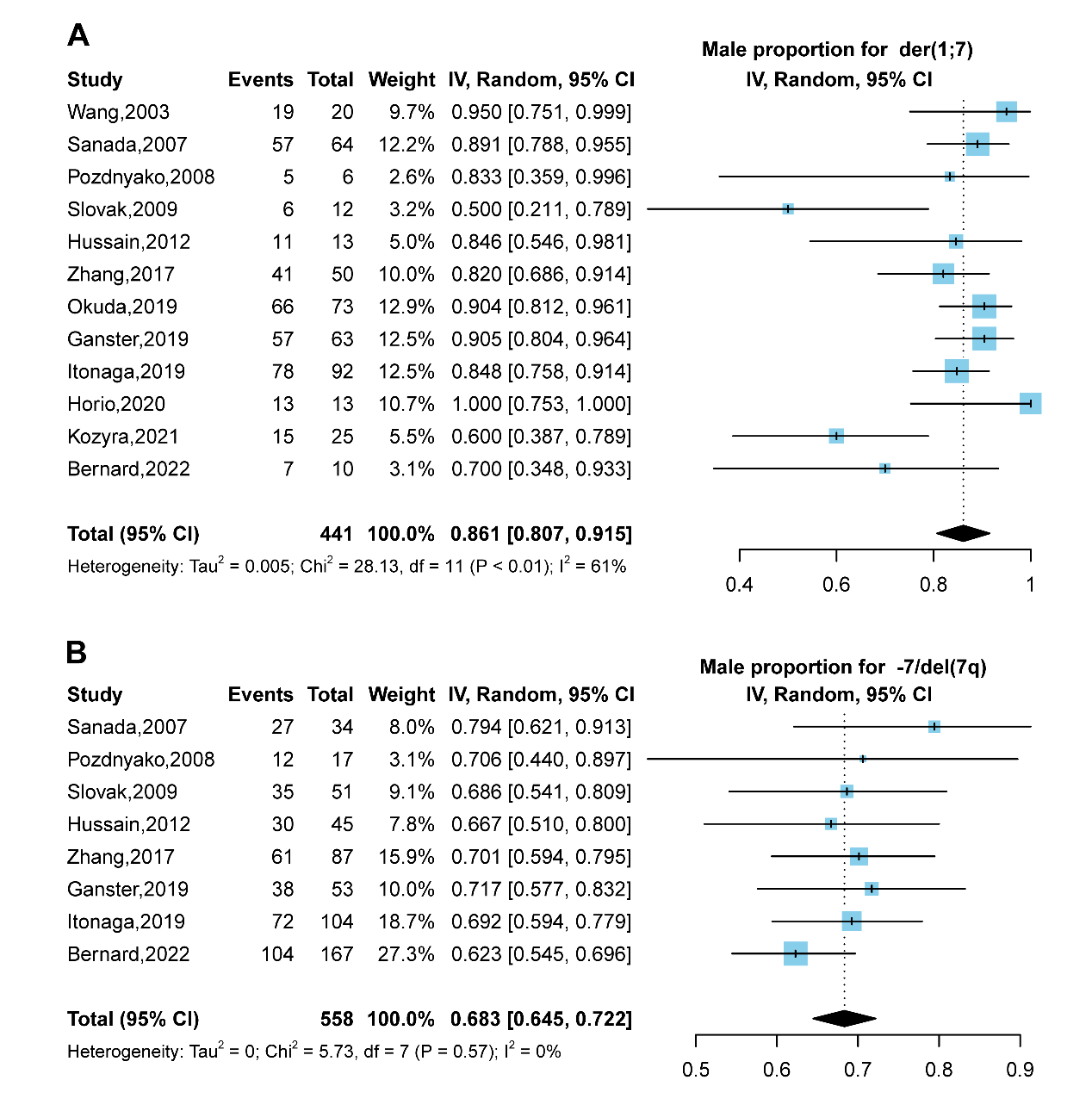


**Supplement Figure S3. The pooled median age at diagnosis for myelodysplastic syndrome patients with der(1;7) and -7/del(7q) abnormalities.** The meta-analysis employs the quantile estimation (QE) method in the 'metamedian' package to calculate the pooled median ages from the extracted median (range) or median (quantile) data.


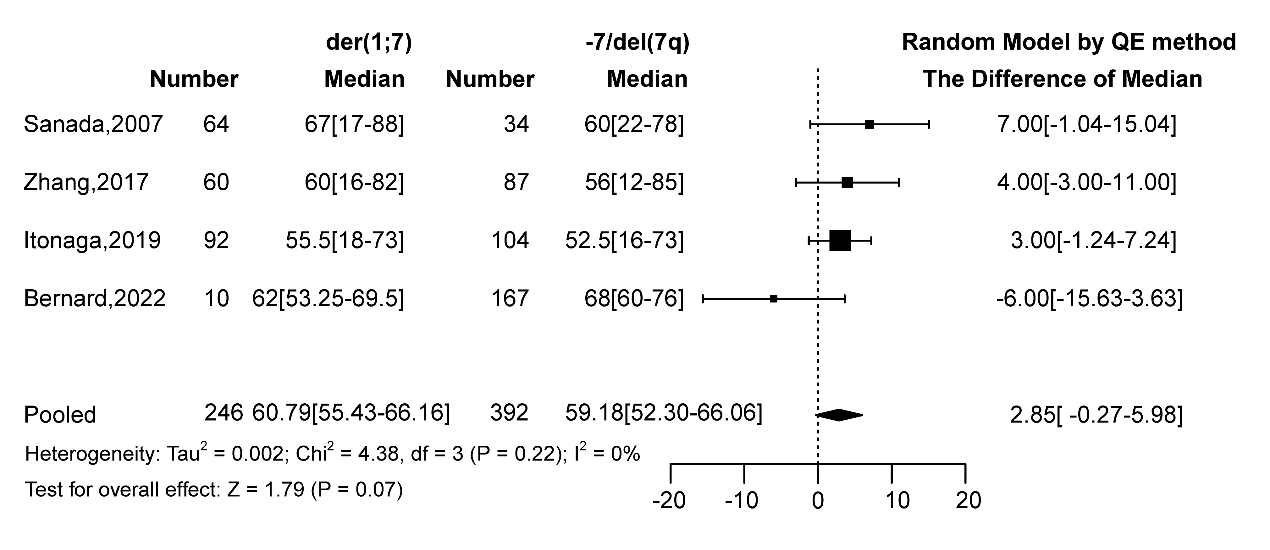


**Supplement Figure S4. The pooled frequencies for myelodysplastic syndrome (MDS) with low blasts in der(1;7) and -7/del(7q) MDS patients.** (A) The frequencies of low blasts MDS (<5% blasts) in MDS with der(1;7). (B) In MDS with -7/del(7q) patients. Both single-arm meta analyses use the random-effect model with the inverse variance method.


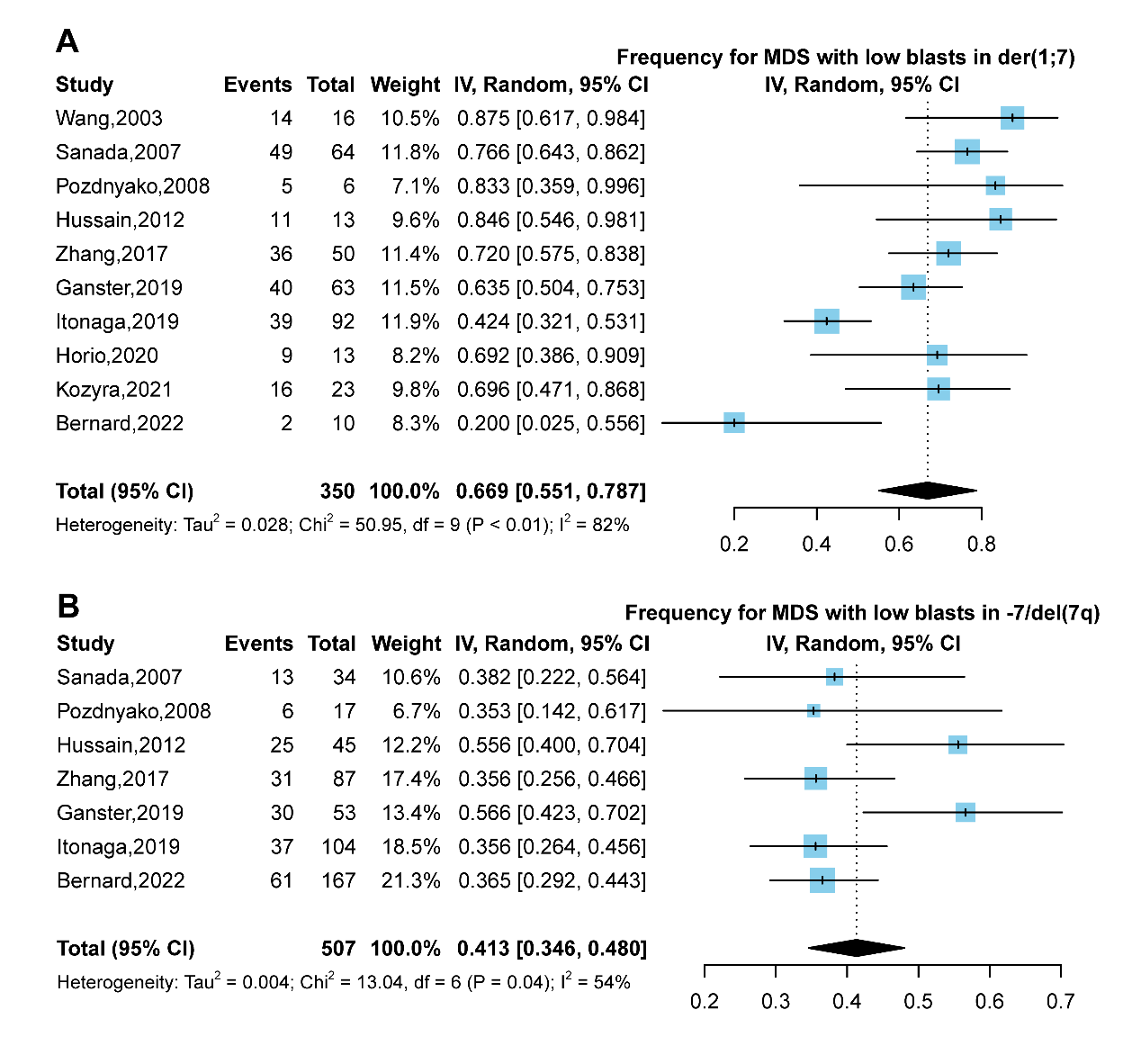


**Supplement Figure S5.** **The pooled frequencies of certain cytogenetic co-aberrations in der(1;7) patients.** (A) der(1;7) as a sole chromosomal abnormality. (B) +8 co-aberration. (C) del(20q) co-aberration. (D) +21 co-aberration. (E) -7 co-aberration. (F) Complex karyotype (CK). (G) -5/del(5q) co-aberration. All single-arm meta-analyses were calculated using the random-effect model with the inverse variance method. CK, complex karyotype.

**
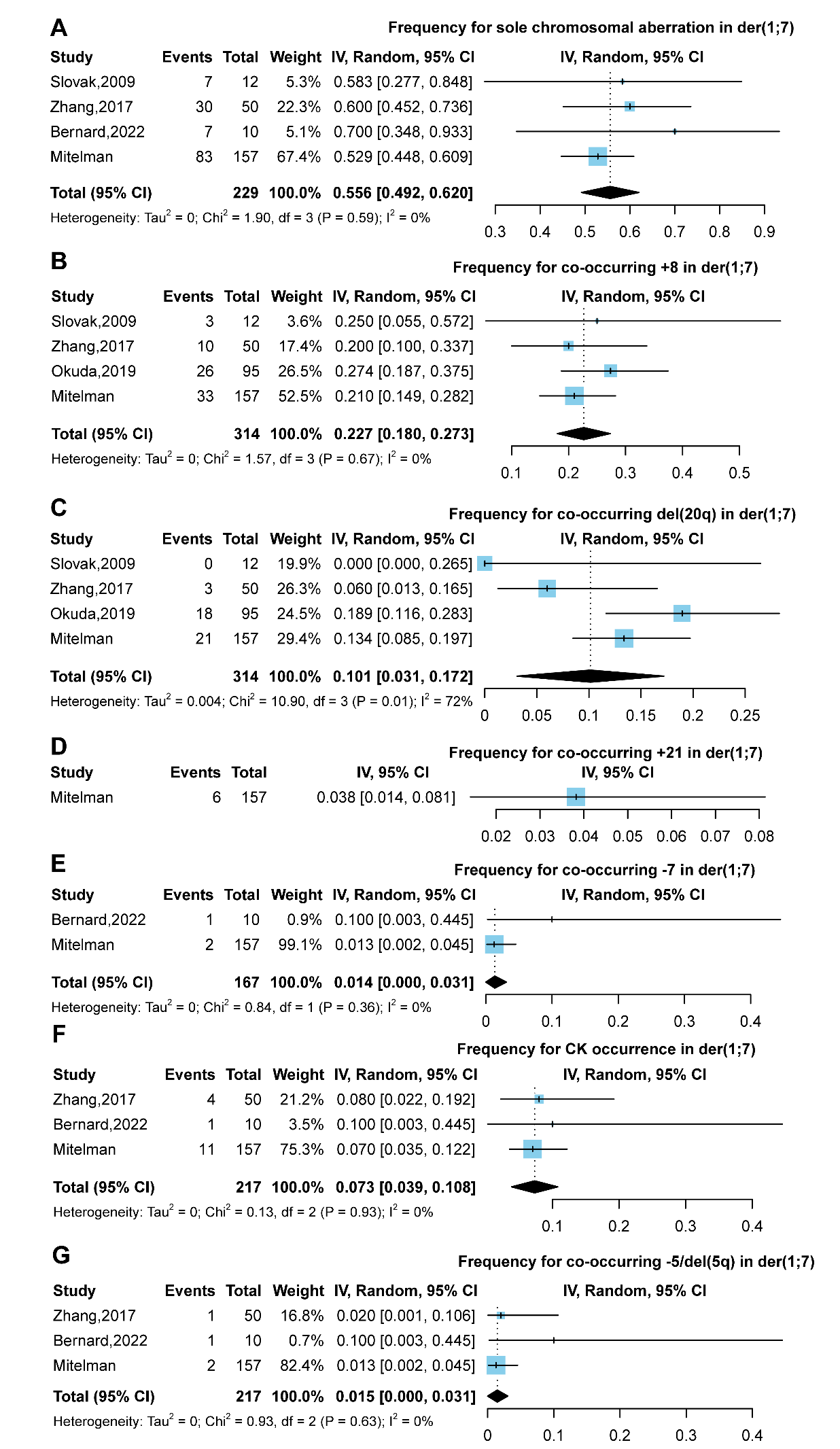
**

**Supplement Figure S6.** **The pooled frequencies of certain cytogenetic co-aberrations in -7/del(7q) patients.** (A) -7/del(7q) as a sole chromosomal aberration. (B) +8 co-aberration. (C) Complex karyotype (CK). (D) -5/del(5q) co-aberration. All single-arm meta-analyses were calculated using the random-effect model with the inverse variance method. CK, complex karyotype.


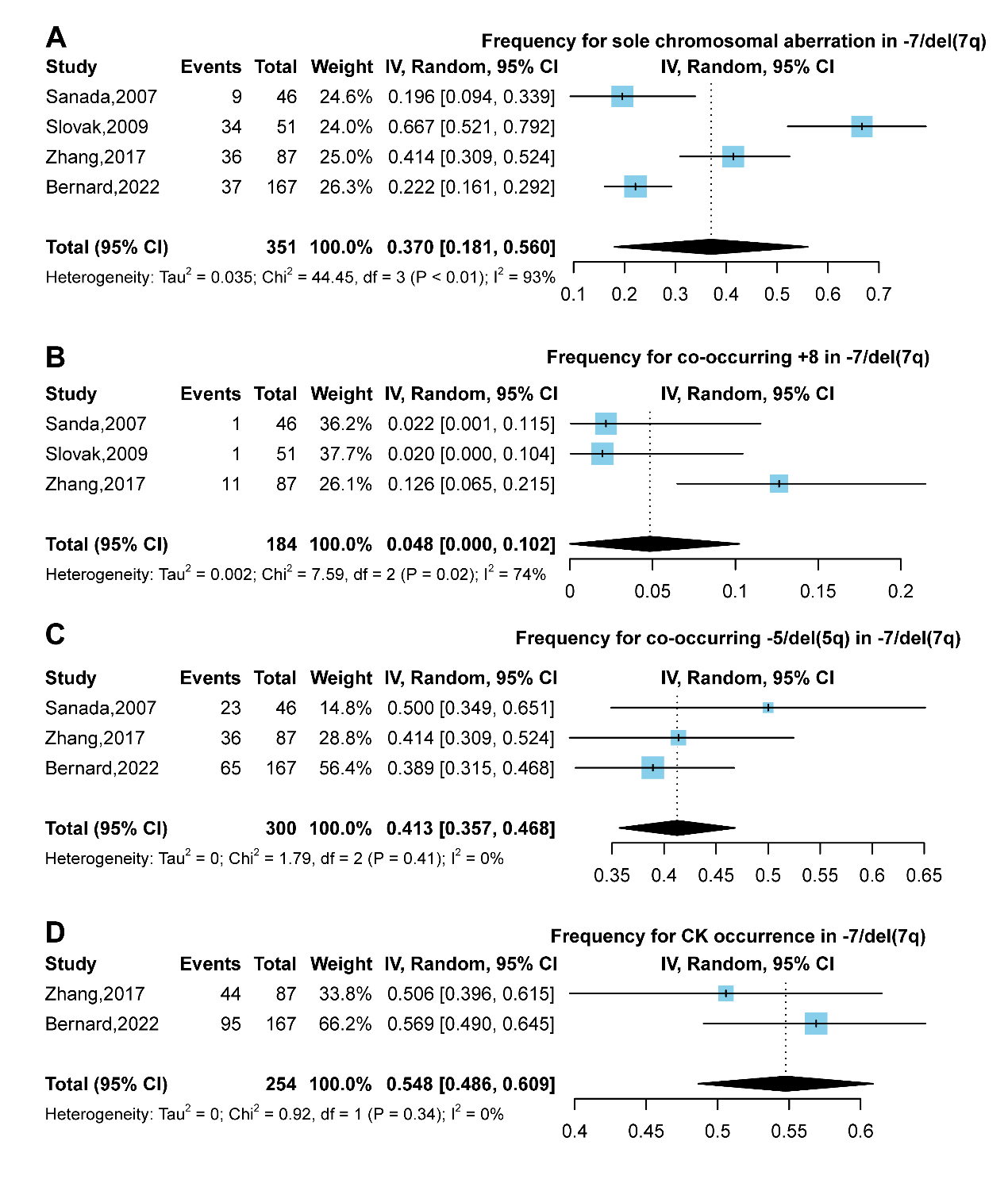


**Supplement Figure S7. The pooled Odds Ratios (ORs) for specific cytogenetic abnormalities co-occurring with der(1;7) compared to -7/del(7q) in myelodysplastic syndrome (MDS) patients.** (A) The pooled ORs for sole chromosomal aberration. (B) +8 co-aberration. (C) Complex karyotype (CK). (D) -5/del(5q) co-aberration. All comparative meta-analyses were performed using random-effects models with a Mantel-Haenszel method.


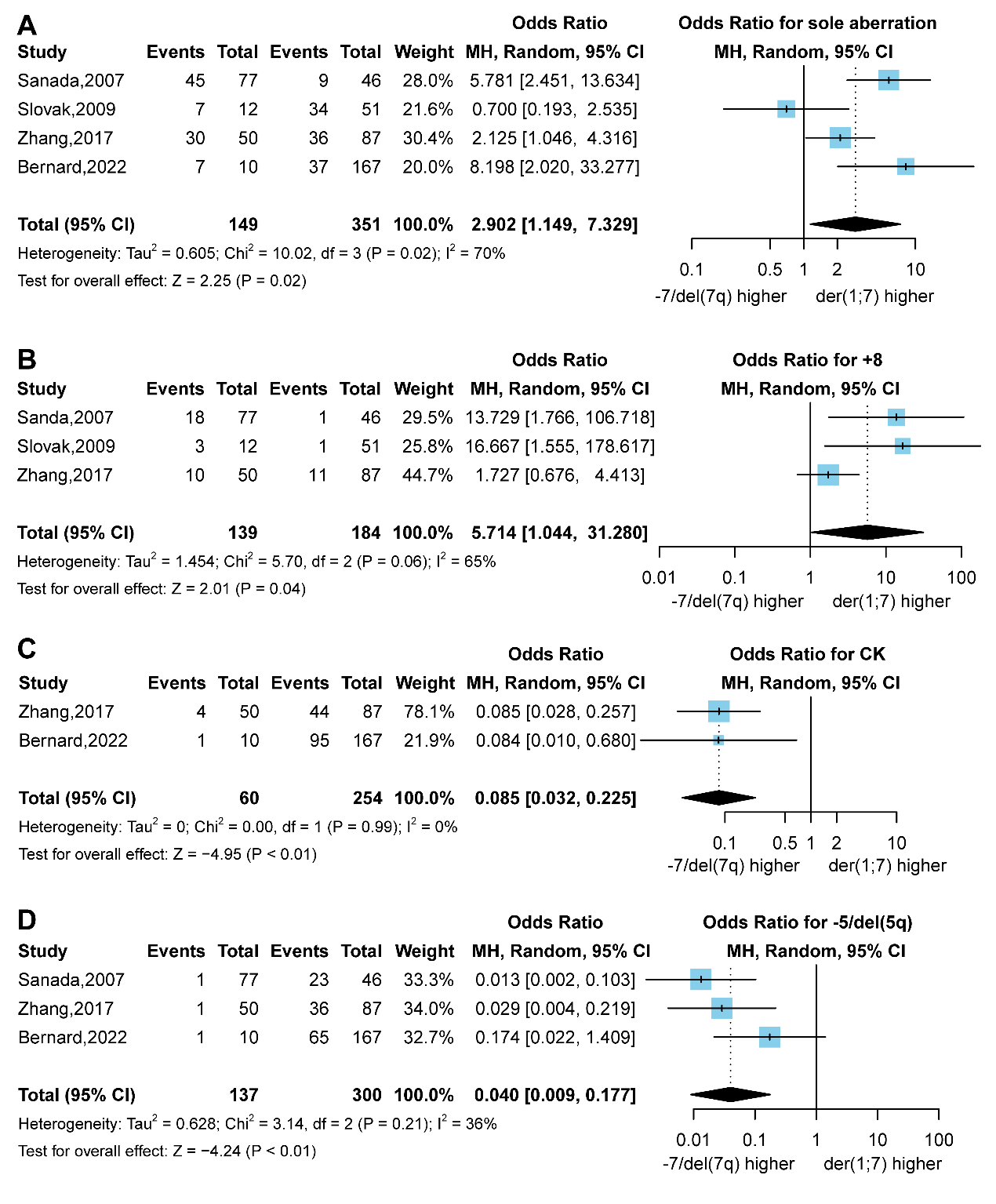


**Supplement Figure S8.** **The frequencies of various gene mutation in MDS with der(1;7) and -7/del(7q) patients**. The data were extracted from published studies, except for Bernard et al.’s study where mutation frequencies and their differences between the two groups were calculated using Fisher's exact test based on individual patient data. The asterisk* denotes a significant difference (P<0.05) in mutation frequencies between der(1;7) and -7/del(7q) groups. Sanger, Sanger’s Sequencing. NGS, Next-Generation-Sequencing. RAS^a^, RAS pathway genes including *CBL*, *KRAS*, *NRAS*, *NF1* and *PTPN11*^1^.


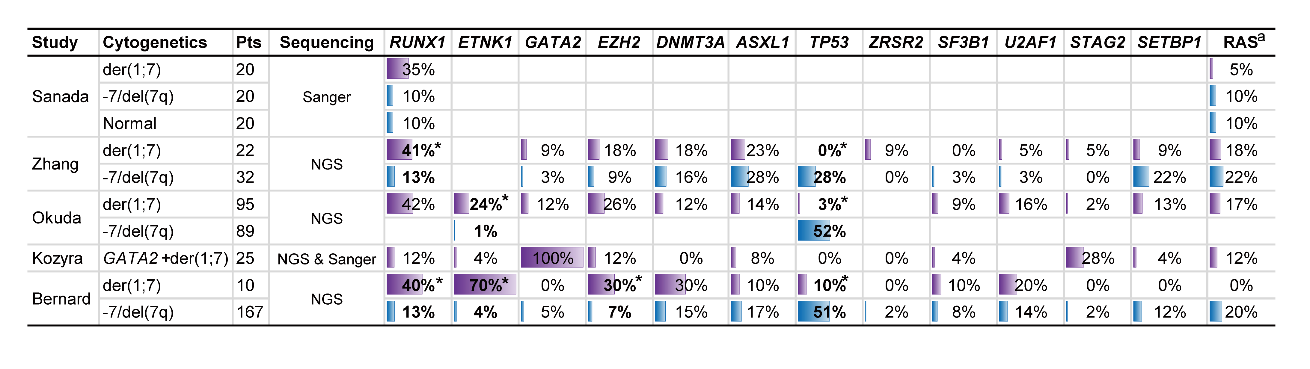


**Supplement Figure S9.** **The pooled frequencies of certain mutations in myelodysplastic syndrome with der(1;7) and -7/del(7q)**. (A) *ASXL1* in der(1;7). (B) *U2AF1* in der(1;7). (C) *STAG2* in der(1;7). (D) *RUNX1* in der(1;7). (E) *RUNX1* in -7/del(7q). (F) *EZH2* in der(1;7). (G) *EZH2* in -7/del(7q). (H) *ETNK1* in der(1;7). (I) *ETNK1* in -7/del(7q). (J) RAS pathway genes in der(1;7). (K) RAS pathway genes in -7/del(7q). (L) *TP53* in der(1;7). (M) *TP53* in -7/del(7q). (N) *GATA2* in der(1;7). (O) *GATA2* in -7/del(7q). (N) *DNMT3A* in der(1;7). (O) *DNMT3A* in -7/del(7q). All meta-analyses were conducted using a random-effects model with inverse variance method, except for *ETNK1* mutation frequencies which were calculated using a fixed effects model and a random effects model.


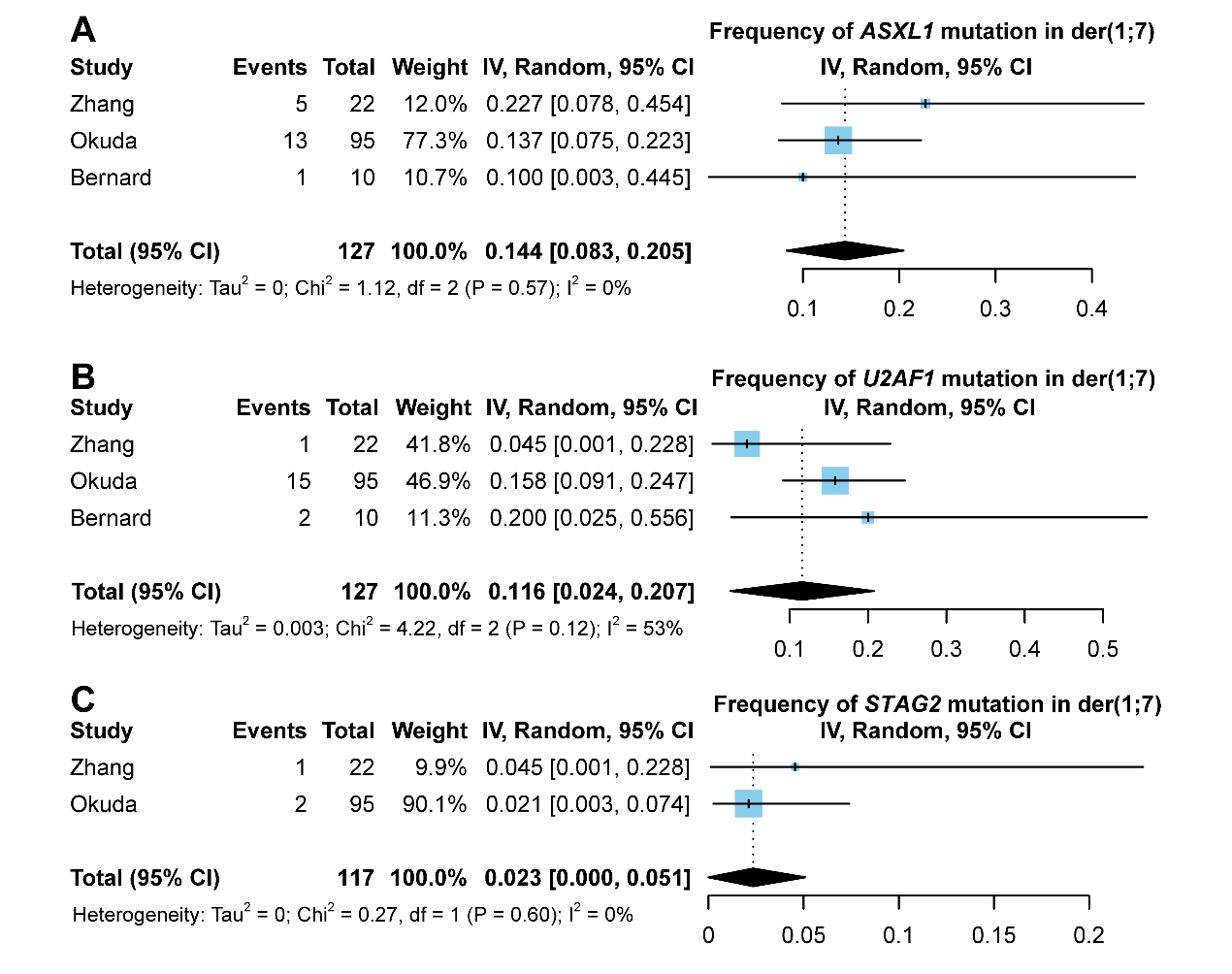


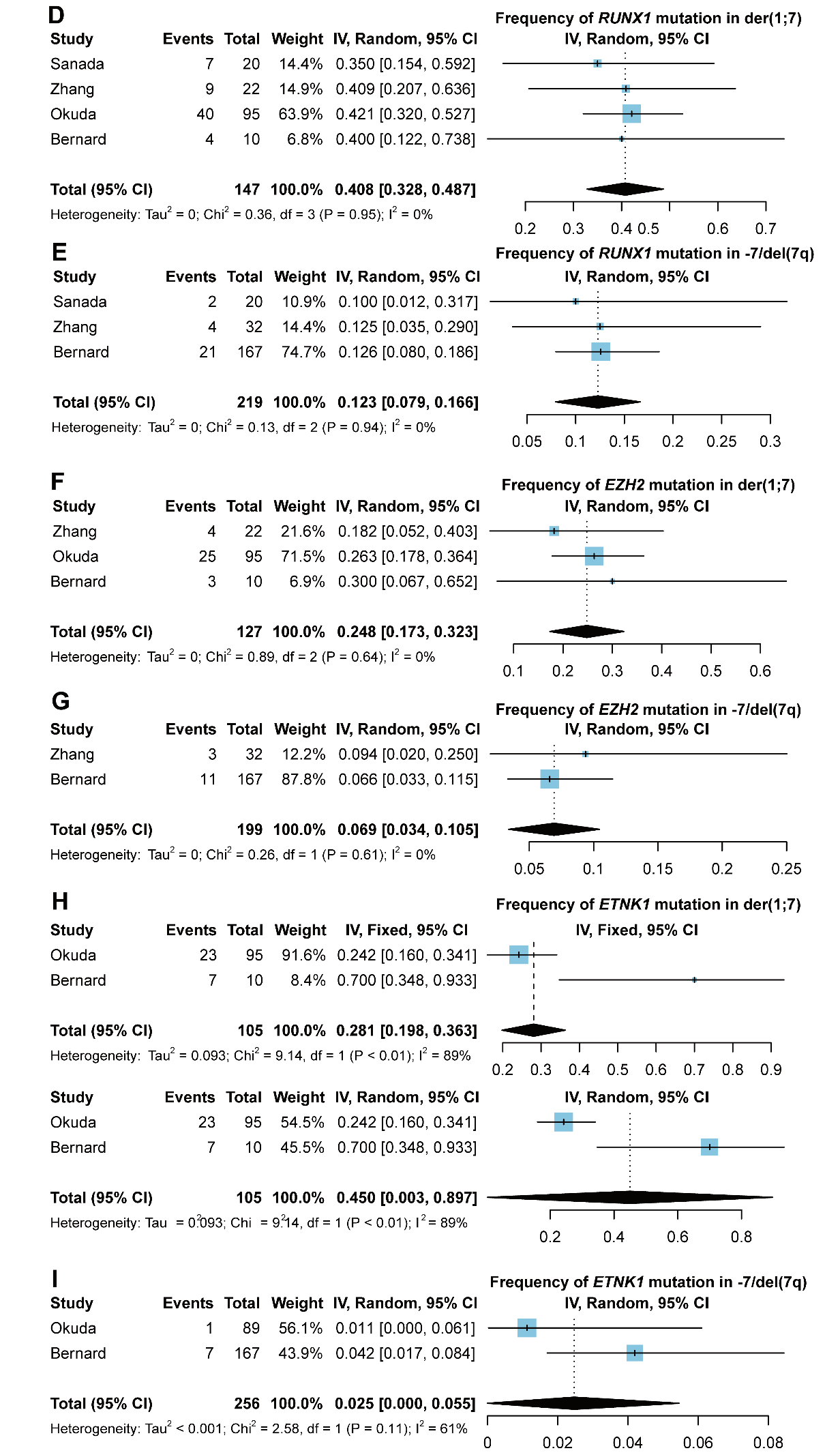


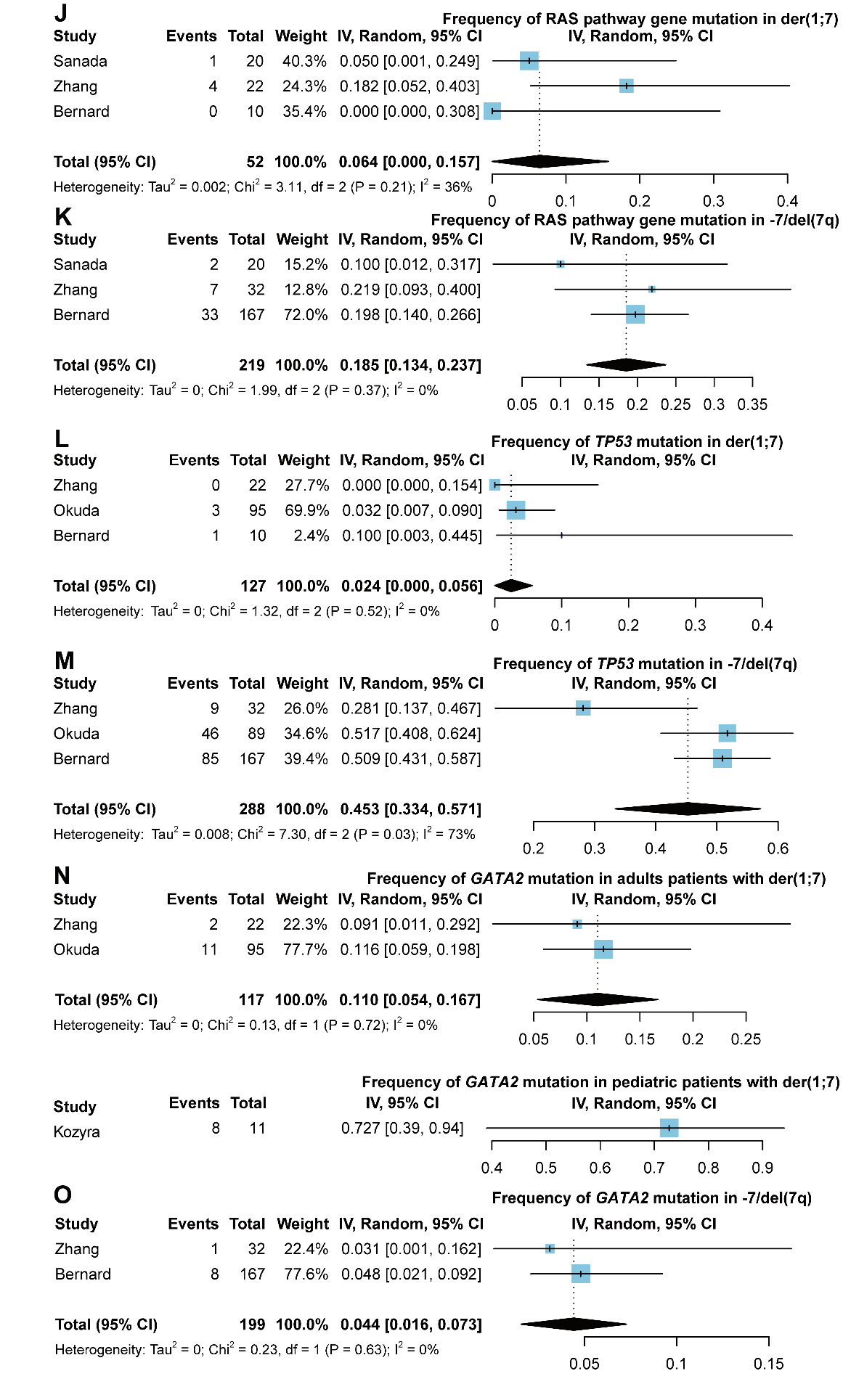


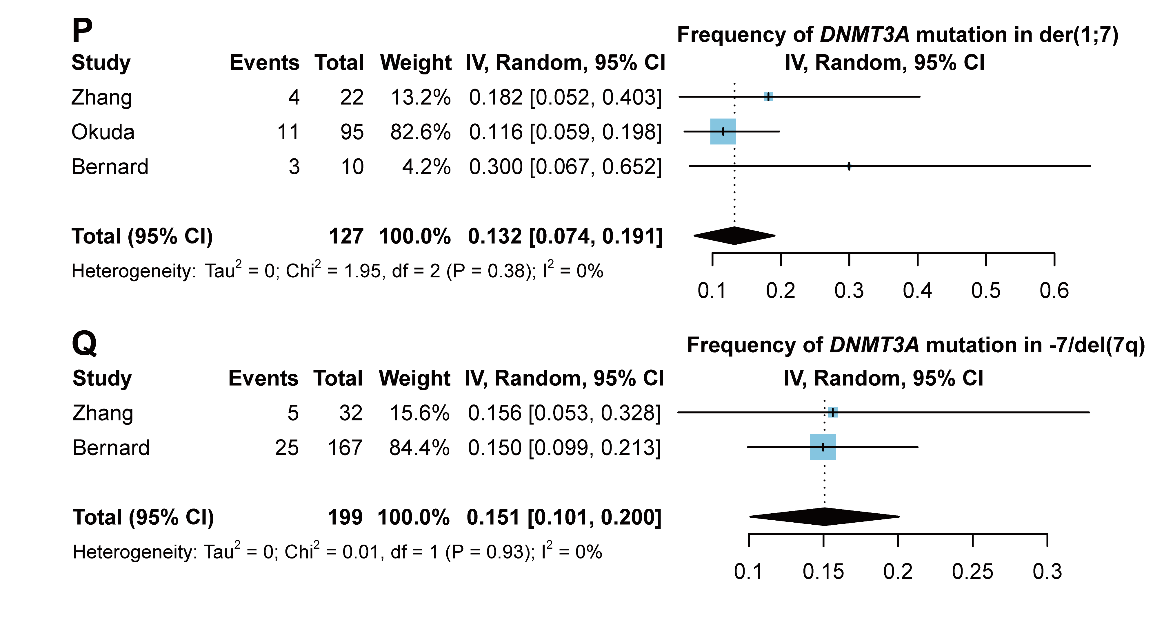


**Supplement Figure S10.** **The pooled Odds Ratios (ORs) for specific gene mutations in myelodysplastic syndrome with der(1;7) versus -7/del(7q).** (A) *RUNX1*. (B) *ETNK1*. (C) *EZH2*. (D) *TP53*. (E) RAS pathway genes. All comparative meta-analyses were conducted using a random-effects model with the Mantel-Haenszel method. An odds ratio and 95% confidence interval above 1 indicate higher odds of certain mutations in der(1;7) patients.


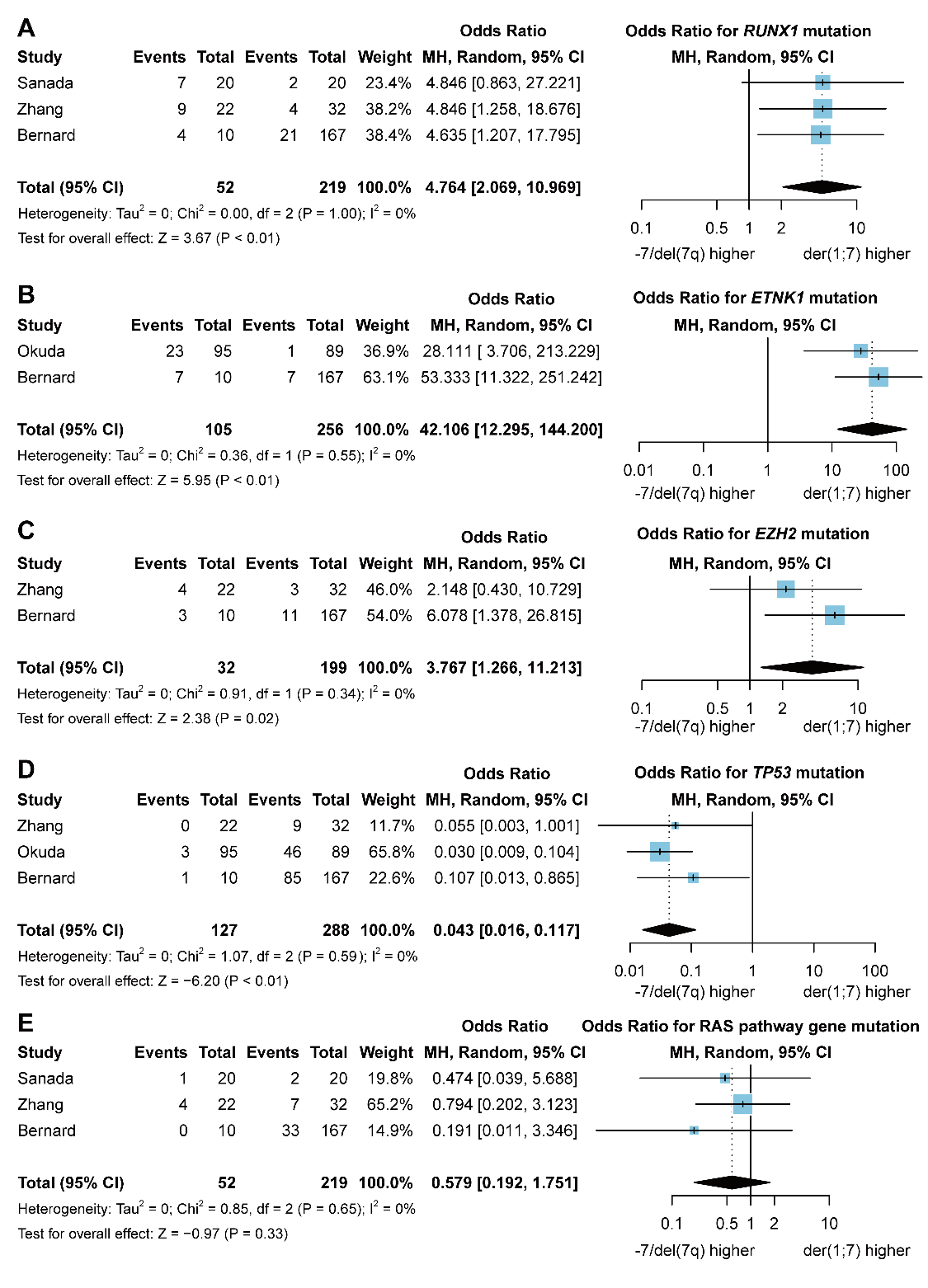


**Supplement Figure S11. The pooled Hazard Ratios (HRs) of** **overall survival (OS) in myelodysplastic syndrome (MDS) patients with der(1;7) compared to those with -7/del(7q)**. The meta-analysis used an inverse variance method with a random-effects model. The pooled HRs and 95% confidence interval are below 1, indicating that der(1;7) patients have significantly better OS compared to -7/del(7q) patients.


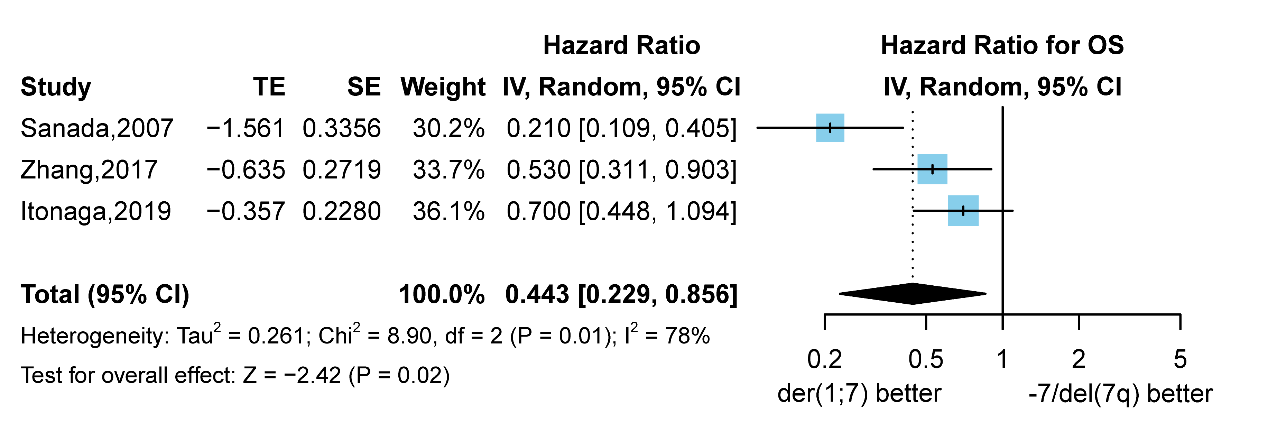


**Supplement Figure S12. The pooled Hazard Ratios (HRs) of overall survival (OS) between sole der(1;7) versus sole -7 MDS patients.** (A) The pooled HRs from two cohorts with sole chromosomal aberration for OS. The random-effects model suggested a favorable but insignificant trend for sole der(1;7), while the fixed-effects model showed better OS for sole der(1;7). (B) The pooled HRs from three cohorts with sole chromosomal aberration for OS. Since -7 patients account for ~80% of -7/del(7q) patients, -7/del(7q) predominantly represents the survival characteristics of -7. After incorporating an additional study (Itonaga et al.’s)^2^ revealed that sole der(1;7) had significantly longer OS and relapse time after transplantation than sole -7/del(7q) patients, both models indicated a better prognosis for sole der(1;7) compared to -7.


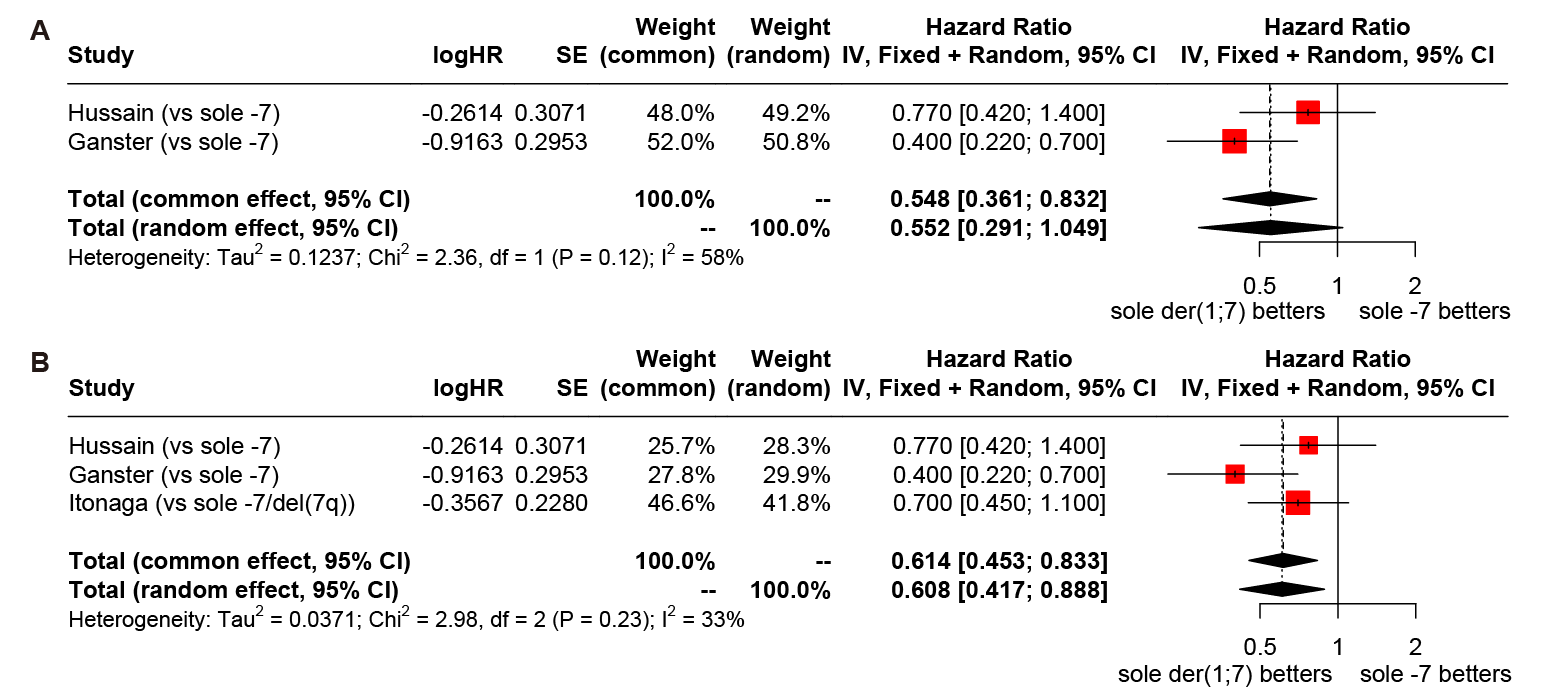


**Supplement Figure S13.** **The pooled Hazard Ratios (HRs) for time to acute myeloid leukemia (AML) progression in myelodysplastic syndrome (MDS) with der(1;7) versus -7 or del(7q)**. The meta-analysis used an inverse variance method with a random-effects model. The pooled HRs and 95% confidence interval are below 1, indicating that der(1;7) patients have a significantly longer time to AML progression compared to -7 or del(7q) patients.


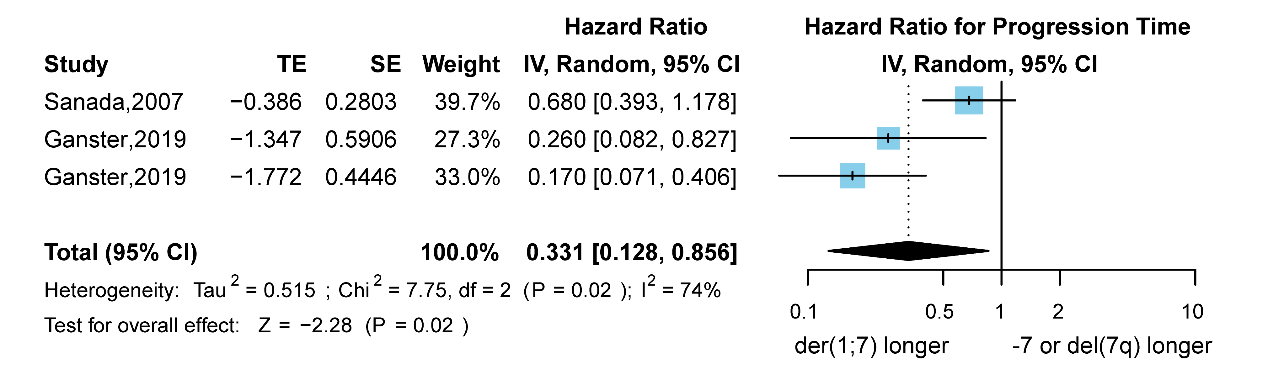


**Supplement Figure S14.** **The** **funnel plots to assess potential publication bias for studies comparing overall survival in myelodysplastic syndrome (MDS) patients with der(1;7) versus -7 or der(1;7) versus del(7q).** (A) The funnel plot for OS in der(1;7) versus -7 patients. (B) The funnel plot for OS in der(1;7) versus del(7q) patients. Each dot represents data from an individual study. The vertical dashed line indicates the pooled HR from the meta-analysis using a random-effects model. The Y-axis represents the standard error of the HR of each study. The roughly symmetrical funnel shape suggests no strong evidence of publication bias for studies comparing these subgroups.


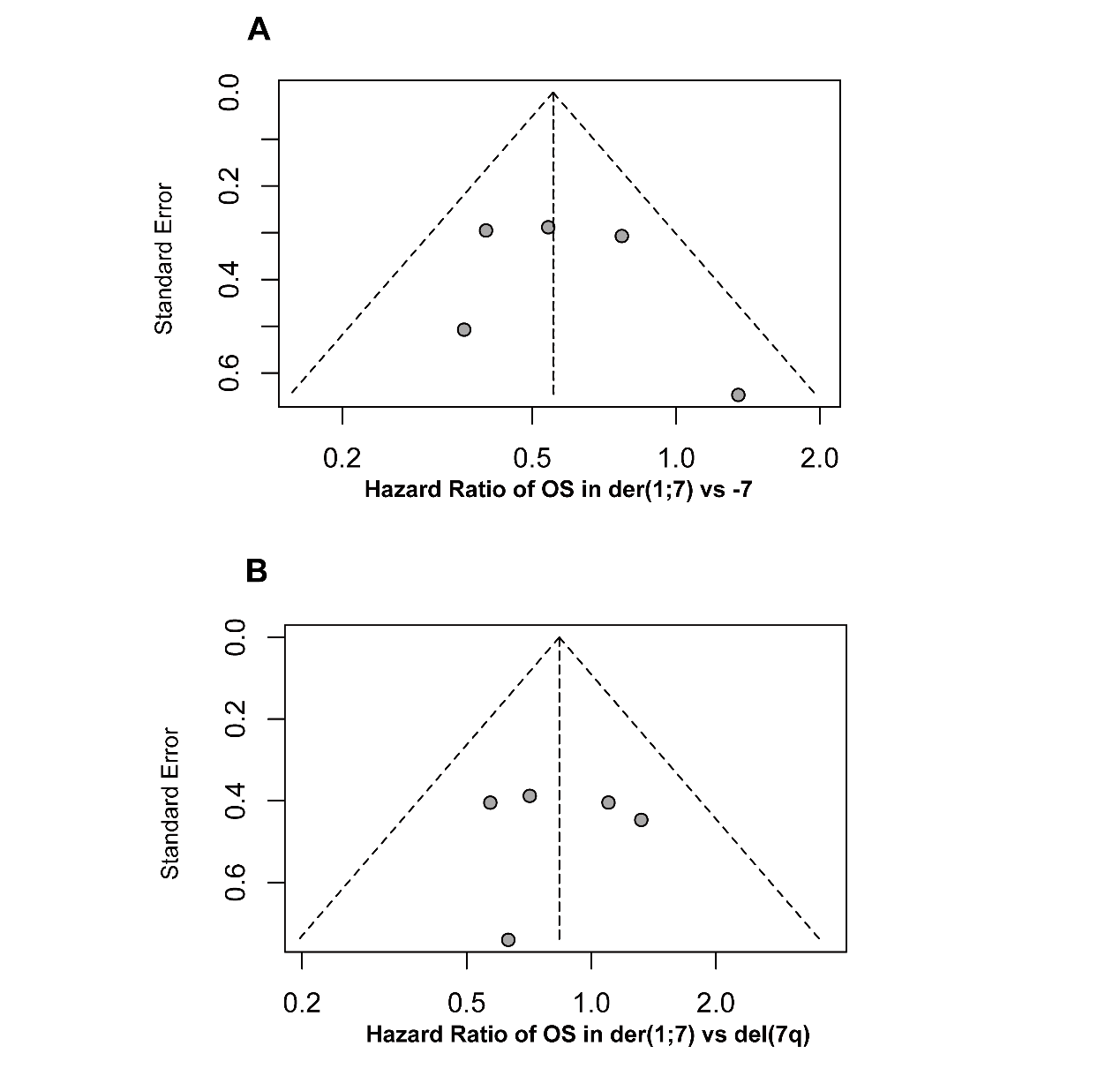


**Supplement Table S1.** The Newcastle-Ottawa Scale (NOS) for assessing the quality and risk bias of studies.

| Studies |  | Selection | | | |  | Comparability |  | Outcome | | |  | Total Quality  score |
| --- | --- | --- | --- | --- | --- | --- | --- | --- | --- | --- | --- | --- | --- |
| Author, year |  | Representativeness of the exposed cohort | Selection of the non- exposed cohort | Ascertainment of exposure | Demonstration that outcome of interest was not present at start of study |  | Comparability and cases of controls |  | Assessment of outcome | Was follow-up long enough for outcomes to occur | Adequacy of follow up of cohorts |  |  |
| Sanada, 2007^3^ |  | 1 | 1 | 1 | 1 |  | 1 |  | 1 | 1 | 1 |  | 8 |
| Pozdnyako, 2008^4^ |  | 1 | 1 | 1 | 0 |  | 1 |  | 1 | 1 | 1 |  | 7 |
| Slovak, 2009^5^ |  | 1 | 1 | 1 | 1 |  | 0 |  | 1 | 0 | 1 |  | 6 |
| Hussain, 2012^6^ |  | 1 | 1 | 1 | 1 |  | 1 |  | 1 | 0 | 1 |  | 7 |
| Ganster, 2019^7^ |  | 1 | 1 | 1 | 1 |  | 1 |  | 1 | 1 | 1 |  | 8 |
| Itonaga,2019^2^ |  | 1 | 1 | 1 | 1 |  | 1 |  | 1 | 1 | 1 |  | 8 |
| Horio, 2020^8^ |  | 1 | 1 | 1 | 0 |  | 1 |  | 1 | 1 | 1 |  | 7 |
| Kozyra, 2021^9^ |  | 1 | 1 | 1 | 0 |  | 1 |  | 1 | 1 | 1 |  | 7 |
| Bernard, 2022^10^ |  | 1 | 1 | 1 | 0 |  | 1 |  | 1 | 0 | 1 |  | 7 |

**Supplement Table S2.** The Egger’s and Begg’s test for publication bias.

|  | Number of comparison pairs | P value by Egger's test | P value by Begg's test |
| --- | --- | --- | --- |
| der(1;7) vs -7/del(7q) |  |  |  |
| Male | 8 | 0.5249 | 0.4579 |
| MDS with low blasts | 7 | 0.602 | 0.6523 |
| Sole karyotype aberration | 4 | 0.9858 | 1 |
| Complex karyotype | 2 | - | - |
| +8 | 3 | 0.0608 | 0.6015 |
| -5/del(5q) | 3 | 0.5139 | 0.6015 |
| *RUNX1* | 3 | 0.7063 | 0.6015 |
| *ETNK1* | 2 | - | - |
| *EZH2* | 2 | - | - |
| *TP53* | 3 | 0.4818 | 0.6015 |
| RAS pathway gene mutation | 3 | 0.2366 | 0.1172 |
| Overall survival | 3 | 0.1374 | 0.1172 |
| der(1;7) vs -7 |  |  |  |
| Overall survival | 5 | 0.6092 | 0.3272 |
| der(1;7) vs del(7q) |  |  |  |
| Overall survival | 5 | 0.8962 | 0.6242 |
| der(1;7) vs -7/-del(7q), -7, or del(7q) |  |  |  |
| Time to progression | 3 | 0.3808 | 0.6015 |

**References**

1. Okuda R, Makishima H, Nannya Y, et al. Distinct, Ethnic, Clinical, and Genetic Characteristics of Myelodysplastic Syndromes with Der(1;7). *Blood*. 2019;134(Supplement_1):5392–5392.

2. Itonaga H, Ishiyama K, Aoki K, et al. Clinical impact of the loss of chromosome 7q on outcomes of patients with myelodysplastic syndromes treated with allogeneic hematopoietic stem cell transplantation. *Bone Marrow Transplant*. 2019;54(9):1471–1481.

3. Sanada M, Uike N, Ohyashiki K, et al. Unbalanced translocation der(1;7)(q10;p10) defines a unique clinicopathological subgroup of myeloid neoplasms. *Leukemia*. 2007;21(5):992–997.

4. Pozdnyakova O, Miron PM, Tang G, et al. Cytogenetic abnormalities in a series of 1029 patients with primary myelodysplastic syndromes: A report from the US with a focus on some undefined single chromosomal abnormalities. *Cancer*. 2008;113(12):3331–3340.

5. Slovak ML, O’Donnell M, Smith DD, Gaal K. Does MDS with der(1;7)(q10;p10) constitute a distinct risk group? A retrospective single institutional analysis of clinical/pathologic features compared to –7/del(7q) MDS. *Cancer Genetics and Cytogenetics*. 2009;193(2):78–85.

6. Hussain FTN, Nguyen EP, Raza S, et al. Sole abnormalities of chromosome 7 in myeloid malignancies: Spectrum, histopathologic correlates, and prognostic implications. *Am. J. Hematol.* 2012;87(7):684–686.

7. Ganster C, Müller‐Thomas C, Haferlach C, et al. Comprehensive analysis of isolated der(1;7)(q10;p10) in a large international homogenous cohort of patients with myelodysplastic syndromes. *Genes Chromosomes Cancer*. 2019;58(10):689–697.

8. Horio T, Enomoto M, Watarai M, et al. Favorable prognostic phenotype in myelodysplastic syndrome with der(1;7)(q10;p10). *eJHaem*. 2020;1(2):558–562.

9. Kozyra EJ, Göhring G, Hickstein DD, et al. Association of unbalanced translocation der(1;7) with germline GATA2 mutations. *Blood*. 2021;138(23):2441–2445.

10. Bernard E, Tuechler H, Greenberg PL, et al. Molecular International Prognostic Scoring System for Myelodysplastic Syndromes. *NEJM Evidence*. 2022;1(7):EVIDoa2200008.
